# Increased phenotypic and functional stability of human allospecific induced Tregs is associated with Vitamin C-mediated *FOXP3* TSDR demethylation

**DOI:** 10.3389/fimmu.2026.1827886

**Published:** 2026-07-08

**Authors:** Evelyn K. Alvarez-Salazar, Judith E. Reyes-Barrientos, Arimelek Cortés-Hernández, Abril Saint-Martin Castellanos, Saúl Arteaga-Cruz, Alejandra Cervera, Iyari Martínez-Iturbe, Beatriz E. Sánchez-Hernández, Armando Gamboa-Domínguez, Gloria Soldevila

**Affiliations:** 1 Department of Immunology, Biomedical Research Institute, National Autonomous University of Mexico, Mexico City, Mexico; 2 National Laboratory of Flow Cytometry, Biomedical Research Institute, National Autonomous University of Mexico, Mexico City, Mexico; 3 Biomedical Genomics and Bioinformatics Laboratory, Instituto Nacional de Medicina Genómica, Mexico City, Mexico; 4 Department of Pathology, Instituto Nacional de Ciencias Médicas y Nutrición Salvador Zubirán, Mexico City, Mexico

**Keywords:** cytokines, demethylation, Foxp3, induced Treg, tolerance, vitamin C

## Abstract

**Introduction:**

While numerous preclinical models emphasize the therapeutic promise of *in vitro*-induced Tregs (iTregs) for managing inflammatory diseases and promoting tolerance, their translation to clinical use is limited by concerns over phenotypic and functional instability, largely due to an epigenetic profile distinct from thymic Tregs.

**Methods:**

To address this, we generated and expanded iTregs in the presence of Vitamin C, known to activate TET enzymes that demethylate critical gene regions such as FOXP3. Antigen-specific Tregs were derived from allogeneic co-cultures of monocyte-derived dendritic cells and naïve T cells. After 7 days, allo-Tregs were isolated by FACS and further expanded for 3 weeks with IL-2, TGF-β, rapamycin, with or without Vitamin C supplementation.

**Results:**

iTregs treated with Vitamin C displayed heightened FOXP3 expression and sustained high levels of suppressive markers (PD-L1, CD39, TIGIT, and CTLA-4), as well as chemokine receptors linked to allograft homing (CCR4, CCR5, and CXCR3). These cells also demonstrated enhanced allospecific suppression of CD4 ^+^ and CD8 ^+^ T cell proliferation, even in the presence of pro-inflammatory cytokines. Notably, this proinflammatory milieu did not trigger the production of intracellular cytokines IL-17 and IFN-γ by allo-iTregs, confirming their sustained phenotypic and functional stability. Advanced analysis demonstrated that allo-iTregs possess a unique profile, setting them apart from conventional T cells. Pyrosequencing of the FOXP3 TSDR revealed reduced CpG methylation in Vitamin C-treated iTregs (60.9% at day 21 and 43.5% at day 28) compared to untreated allo-iTregs (85.9% at day 21 and 80.5% at day 28) and naïve T cells (92.8%). In addition, transcriptomic analysis demonstrated that the core Treg transcriptional signature remained largely intact with Vitamin C treatment, irrespective of cytokine exposure, whereas the major transcriptional changes were associated with activation, proliferation, and cell cycle regulation.

**Discussion:**

In summary, Vitamin C enhances both the phenotypic and functional stability of allospecific iTregs, even under proinflammatory conditions, correlating with increased TSDR demethylation, while preserving the transcription of key Treg genes. These results suggest that Vitamin C-treated allospecific iTregs are superior candidates for immunotherapy strategies to promote long-term tolerance in transplant recipients.

## 1 Introduction

Currently, conventional therapies aimed at preventing organ/tissue rejection and to treat autoimmune and inflammatory diseases are based mainly on administration of nonspecific immunosuppressive drugs. However, their lack of selectivity is accompanied by numerous side effects such as opportunistic infections, neoplasias and systemic toxicity, which progressively compromise patient’s quality of life and survival (1). For this reason, the development of specific and effective immunotherapies capable of directly targeting pathogenic immune cells may in future replace, at least in part, generalized immunosuppressive drug regimens (2). In this context, adoptive regulatory T cell therapy has emerged as a promising alternative to conventional treatments, exhibiting a favorable safety profile and logistical feasibility in early-phase human clinical trials (3, 4).

Regulatory T cells (Tregs) are CD4^+^ T cells critical for the maintenance of immune homeostasis and the control of immune pathologies. They are defined by the expression of the transcription factor Foxp3 and can be divided into two major subpopulations based on their origin: thymic Tregs (tTregs) and peripheral Tregs (pTregs). Peripheral Treg cells arise from naïve T cells and can be generated in two ways: physiologically (known as pTregs) or *in vitro* (known as iTregs) (5). Additionally, other T CD4^+^FOXP3^-^ cells with suppressor capacity have been described, such as Tr1 and Th3 cells, which secrete IL-10 and TGFβ, respectively (6). Generation of iTregs is associated with suboptimal TCR signaling and the presence of TGF-β, and IL-2, which promote *de novo* Foxp3 expression in conventional T cells. Like tTregs, iTregs also express immunoregulatory markers and have suppressive capabilities (7).

Evidence from experimental animal models of inflammation, transplantation and autoimmunity support a critical role for iTregs in regulating immune tolerance and disease expression (8, 9). Both *in vitro* and *in vivo* studies demonstrate that iTregs are particularly effective at suppressing acute inflammation as a result of their enhanced ability to recognize inflamed endothelium (10). Moreover, iTregs exhibit increased resistance to Th17 conversion in response to IL-6 both *in vitro* and *in vivo* (11) and, in contrast to tTregs, their differentiation, function and stability were not affected by high salt environments (12), and were more effective at controlling disease progression than tTregs in a model of autoimmune gastritis (13), suggesting a greater adaptability to inflammatory and metabolic stress conditions. However, despite robust immune tolerance observed in preclinical models, achieving definitive and sustained operational tolerance in clinical settings remains a significant challenge. Reproducible clinical efficacy in humans is constrained by difficulties in maintaining Treg survival, homing, phenotypic, and functional stability within hostile, pro-inflammatory post-transplant environments (14, 15).

Stable Treg identity is established during thymic development through epigenetic mechanisms, particularly DNA demethylation at Treg-specific genomic loci (FOXP3, CTLA4, IKZF2, IL2RA, TNFRSF18), as well as the formation of super-enhancers (16, 17). Notably, TCR stimulation-induced epigenetic changes and FoxP3 expression represent independent and complementary events required for Treg cell lineage specification (18). Maintenance of this epigenetic landscape is critical for preserving Treg suppressive function and preventing the acquisition of pro-inflammatory phenotypes. Several strategies have been explored to enhance iTreg stability, including the use of mTOR pathway inhibitors such as rapamycin, stabilizing agents like retinoic acid (19), histone-modifying compounds such as butyrate (20), activators of Ten–Eleven Translocation (TET) enzymes (21), inhibition of cyclin-dependent kinases and CD28 signaling pathway (22, 23) and genetic engineering approaches including CRISPR-Cas technologies (24, 25), – all aimed at enhancing stable iTreg phenotype and function for immunotherapy.

Vitamin C has been shown to enhance the activity of TET enzymes, which mediate the initiation and maintenance of hypomethylation at Treg-specific regions, including CNS1, CNS2, and cis-regulatory elements within the *FOXP3* locus (21, 26). TET enzymes oxidize 5-methylcytosine (5mC) to 5-hydroxymethylcytosine, an intermediate product of active DNA demethylation. Vitamin C supplementation during iTreg generation enhances TSDR demethylation of the *FOXP3* gene and consequently stable FOXP3 expression as well as suppressive function *in vitro* and *in vivo*. Furthermore, hypoxia enhances these effects by activating different signaling and metabolic pathways compared to standard normoxic conditions (27).

Although the capacity of Vitamin C to potentiate TET-mediated *FOXP3* demethylation is well established, conventional protocols have primarily focused on murine models, short-term cell cultures, or non-specific cell stimulation (3, 27, 28). The feasibility of incorporating Vitamin C into large-scale manufacturing platforms to maintain the lineage stability of human antigen-specific iTregs has not yet been addressed. To bridge this translational gap, we previously demonstrated that human allospecific iTregs can be efficiently expanded *in vitro* while preserving their suppressive phenotype and function (29). However, prolonged expansion of allo-iTregs led to increased methylation of the *FOXP3* TSDR region, potentially compromising lineage stability and therapeutic efficacy (30). To address this challenge, the present study investigates the impact of Vitamin C supplementation on the large-scale production of human allo-iTregs derived from naïve T cells and assesses their phenotype, suppressive function, epigenetic status, and transcriptomic profile in the presence of pro-inflammatory cytokines.

Our results indicate that incorporating Vitamin C into large-scale expansion protocols effectively reduces progressive TSDR methylation, resulting in allo-iTregs with improved phenotypic and functional stability under pro-inflammatory conditions. Collectively, these findings establish a scalable, clinically translatable platform for generating epigenetically stable, antigen-specific Treg therapies that promote durable immunological tolerance in transplant recipients.

## 2 Materials and methods

### 2.1 Sample collection

Buffy coats derived from peripheral blood were obtained from the Blood Bank of the Instituto Nacional de Ciencias Médicas y Nutrición Salvador Zubirán. These leukocyte concentrates, collected from healthy **male** donors with written informed consent, complied with institutional regulations and current ethical guidelines. Peripheral blood mononuclear cells (PBMCs) were isolated from these concentrates by density gradient centrifugation using Ficoll-Hypaque. A fraction of the PBMCs was cryopreserved at −70 °C and then stored in liquid nitrogen, while the remaining cells were used immediately for subsequent culture assays.

### 2.2 Reagents and antibodies

The following reagents were used for cell culture assays: Dynabeads™ Human T-Activator CD3/CD28 beads, CTS OpTmizer™ T Cell Expansion medium, RPMI culture medium, fetal bovine serum (FBS), CellTrace™ Yellow (CTY), and CellTrace™ Violet (CTV) Proliferation Kit, all from Thermo Scientific (Waltham, MA, USA). Human AB serum was obtained from Biowest (Nuaillé, France). All-trans retinoic acid (ATRA), rapamycin, Ficoll-Paque™, and Vitamin C (L-ascorbic acid) were obtained from Sigma-Aldrich (St. Louis, MO, USA). Recombinant human cytokines TGF-β1, IL-2, IL-4, GM-CSF, IL-6, IL-1β, IFN-γ, and TNF-α were purchased from PeproTech (Cranbury, NJ, USA). All culture media were supplemented with L-glutamine, sodium pyruvate, non-essential amino acids, and antibiotic–antimycotic solution (containing penicillin, streptomycin, and an antifungal agent).

For flow cytometry analyses, the following antibodies were used: anti-CD45RA APC-Fire™ 750, Zombie Aqua™ Fixable Viability Kit, anti-CD4 BV605, anti-CD25 PE, anti-CD70 PE-CF594, anti-CD39 BV711, anti-CTLA-4 Brilliant Violet™ 421, anti-FOXP3 Alexa Fluor™ 647, anti-CXCR3 Brilliant Violet™ 711, anti-CCR7 PerCP-Cy5.5, anti-CCR4 Brilliant Violet™ 421, and anti-CCR2 Brilliant Violet™ 605 (all from BioLegend, San Diego, CA, USA). Anti-CD3 APC, anti-CD27 PE-Cy7, anti-CD4 APC-Cy7, anti-CD8 PE-Cy7, and anti-CD14 PE-Cy7 were obtained from Tonbo Biosciences (San Diego, CA, USA).

### 2.3 Coculture between monocyte-derived dendritic cells and naïve T cells

CD14+ monocytes were obtained from PBMCs by positive selection using magnetic MicroBeads according to the manufacturer’s protocol. The isolated CD14^+^ cells were cultured for 8 days in RPMI medium supplemented with 10% human AB serum, 50 ng/ml GM-CSF, and 50 ng/ml IL-4 in 48-well plates to differentiate into Monocyte-derived dendritic cells (Mo-DCs). At the end of the differentiation period, Mo-DCs were harvested, washed, and irradiated with 3,000 rads prior to use in functional assays.

Naïve T cells were isolated from PBMCs of an unrelated healthy donor (allogeneic) by staining with anti-CD4, anti-CD25, and anti-CD45RA monoclonal antibodies for 20 minutes at 4 °C in the dark. Cells were then washed and resuspended in 1x PBS. Naïve T cells were sorted based on a CD4^+^CD25^-^CD45RA^+^ gating strategy on a FACSAria I cell sorter (BD Biosciences). Sorted cells were collected in RPMI medium supplemented with 20% fetal bovine serum (FBS) to maintain cell viability and membrane integrity during sorting-induced mechanical stress. Subsequently, cells were washed twice with 1x PBS, stained with CellTrace Violet (CTV), and resuspended in CTS OpTmizer T-Cell Expansion culture medium.

CTV-labeled naïve T cells were co-cultured with irradiated allogeneic Mo-DCs at a ratio of 10:1 (T cells to Mo-DCs) for 7 days in 96-well U-bottom plates. The co-culture medium was supplemented with 5 ng/mL TGF-β1, 100 U/mL IL-2, 10 nM ATRA, and 1% human AB serum.

### 2.4 Isolation and expansion of induced allospecific Tregs

On day 7 of co-culture, cells were stained with anti-CD4, anti-CD25, and Zombie Aqua™ viability dye for 20 minutes at room temperature in the dark and then washed with 1x PBS. Proliferating T cells (CD4^+^CD25^+^^+^CTV^-^) were isolated using a FACSAria I cell sorter. Sorted cells corresponding to allospecific iTregs (allo-iTregs) were collected in RPMI medium supplemented with 20% FBS, washed twice, resuspended in CTS™ OpTmizer™ T-Cell Expansion medium containing only 50 U/mL IL-2, and cultured for 3 days.

After the initial 3-day culture, cells were washed and then stimulated with anti-CD3/CD28-coated beads in the presence of 200 U/mL IL-2, 5 ng/mL TGF-β, and 100 ng/mL rapamycin, with or without Vitamin C (20μg/mL) for 4 days (expansion phase). After this expansion phase, the beads were removed, and cells were washed once before being cultured for an additional 3 days in medium supplemented with only 50 U/mL IL-2 (resting phase). This expansion and resting cycle was then repeated weekly for three consecutive weeks. To further evaluate the stability of allo-iTregs, 10 ng/mL recombinant human IL-1β, IL-6, and TNF-α pro-inflammatory cytokines were added to the culture during the second week of expansion, following the described expansion and resting protocols. **Supplementary Figure 1** presents a comprehensive visual representation of the protocol used to induce and expand alloantigen-specific iTregs.

### 2.5 Surface and intracellular staining

Allospecific iTregs were phenotypically characterized by flow cytometry using fluorochrome-conjugated monoclonal antibodies targeting surface markers (CD4, CD25) and the intracellular transcription factor FOXP3. Co-inhibitory (PD-L1, CD39, CD73, TIGIT, CTLA-4. TIM-3) and chemokine receptors (CCR4, CCR5, CCR7, CXCR3) were also evaluated. Surface staining followed previous procedures. For intracellular staining, cells were first labeled on the surface, then permeabilized with fixation/permeabilization solution (Tonbo Biosciences), and incubated for 12 h at 4 °C. After washing with 1x permeabilization buffer, cells were stained with anti-FOXP3 Alexa Fluor^®^ 647–conjugated antibody for 30 min at 4 °C, then washed again and acquired on Attune^®^ NxT flow cytometer (Life Technologies). Data were analyzed using FlowJo v11 (BD) and GraphPad Prism v8 softwares. Prior to immunophenotypic analysis, raw flow cytometry data underwent quality control preprocessing using the PeacoQC algorithm plugin in FlowJo, to systematically eliminate acquisition artifacts and flow rate anomalies. Subsequently, high-quality data (“good events”) were subjected to the sequential gating hierarchy detailed in **Supplementary Figure 2**, to identify and analyze the populations of interest.

### 2.6 *In vitro* suppression assay

At week 3 of the allo-iTreg expansion protocol, the suppressive function of the cells was evaluated by measuring their capacity to inhibit T-cell proliferation. Allogeneic or third-party monocyte-derived dendritic cells (Mo-DCs) were generated 8 days prior to the suppression assays, as previously described.

Peripheral blood mononuclear cells (PBMCs) from the same donor used for iTreg generation were thawed. Autologous CD3^+^ responder T cells (Tresp) were isolated using a Pan T Cell Isolation Kit (Miltenyi Biotec) according to the manufacturer’s instructions. The isolated CD3^+^ T cells were labeled with CellTrace™ Yellow (CTY) and co-cultured with allospecific iTregs previously stained with CTV at various Treg: Tresp ratios (0:1, 1:3, 1:9, and 1:27). Co-cultures were stimulated with allogeneic or third-party Mo-DCs at a Mo-DC:T-cell ratio of 1:2.

After 4 days of co-culture, cells were stained with a viability marker and anti-CD4 and anti-CD8 monoclonal antibodies for 20 minutes at 4 °C in the dark. Following staining, cells were washed twice and acquired on a flow cytometer. Proliferation of CD4^+^ and CD8^+^ responder T cells was quantified by CTY dilution after exclusion of CTV^+^ iTregs. Suppressive activity was determined as the inhibition percentage in responder T cell proliferation using the following formula:

[(Proliferation of Tresp without Tregs – Proliferation of Tresp with Tregs)/Proliferation of Tresp without Tregs] × 100.

### 2.7 Intracellular cytokine production assay

On day 21 of allospecific iTreg expansion, an intracellular cytokine staining assay was performed to assess pro-inflammatory cytokine production. Cells were collected, washed and then incubated for 4 hours in RPMI medium with 10% FBS, 40 ng/mL PMA, 400 ng/mL ionomycin, and 1 µg/mL brefeldin A (GolgiPlug™, BD Biosciences).

After incubation, cells were stained with anti-CD4 APC-Cy7 and Zombie Aqua™ viability dye for 15 minutes. Next, cells were fixed and permeabilized with 100 µL fixation/permeabilization solution for 1 hour, then blocked with 1 µL Human TruStain FcX™ in 19 µL permeabilization buffer for 10 minutes at 4 °C. Intracellular staining was performed using antibodies against IFN-γ (PE-Cy7), IL-17A (Alexa Fluor 488), T-bet (BV711), ROR-γt (BV421), and FOXP3 (Alexa Fluor 647) for 30 minutes at 4 °C. Finally, cells were washed with a permeabilization buffer and acquired on Cytoflex LX flow cytometer.

### 2.8 *FOXP3* TSDR DNA methylation analysis

The methylation status of the *FOXP3* Treg-specific demethylated region (TSDR) was evaluated in allo-iTregs before and after three weeks of expansion. Naïve T cells (CD4^+^CD25^-^CD45RA^+^) and thymic Tregs (CD4^+^CD25^++^CD127^lo/-^) were isolated from healthy donor PBMCs using a FACSAria I cell sorter and used as controls. DNA methylation of the TSDR was analyzed by pyrosequencing using specific primers designed with PyroMark v2.0 software (QIAGEN Inc., Hilden, Germany). Briefly, 0.5 µg of genomic DNA was modified with sodium bisulfite using the EZ DNA Methylation-Direct Kit (Zymo Research Corp., Irvine, CA) according to the manufacturer’s instructions. PCR amplification was carried out in a total volume of 15 µL using the PyroMark PCR Kit (QIAGEN) with 2 µL of bisulfite-converted DNA as a template. The amplified product was incubated with streptavidin-coated Sepharose beads (Cytiva, Marlborough, MA, USA) in binding buffer for 15 minutes under constant agitation, followed by sequential washes with 70% ethanol and denaturation using PyroMark denaturing solution (QIAGEN). Subsequently, hybridization was performed using a specific sequencing primer diluted in alignment buffer (QIAGEN) for 2 minutes at 80 °C. Pyrosequencing was conducted using the PyroMark Q24 enzyme and substrate mix on a PyroMark Q24 system, and data were analyzed using the PyroMark Q24 Software (QIAGEN). Primer sequences used for amplification and sequencing are listed in **Supplementary Table 1**.

### 2.9 RNA-seq analysis

Total RNA was extracted from *ex vivo* expanded T cells using the PureLink™ RNA Mini Kit, following the manufacturer’s instructions. RNA integrity and purity were assessed by spectrophotometry and agarose gel electrophoresis prior to library preparation. High-quality RNA samples were submitted to Novogene (Beijing, China) for library construction and sequencing. Libraries were generated according to standard protocols and sequenced on the Illumina NovaSeq X Plus System, yielding 150 bp paired-end reads. Raw data underwent standard quality control using FastQC (v0.12.1) and fastp (v0.23.4) (31) for adapter trimming, low-quality read filtering, and GC content assessment. Transcript-level quantification was performed using Salmon (v1.10.3) (32) using Ensembl GRCh38 human reference and gene annotation version 113. Estimated counts at the transcript level were aggregated to the gene level with tximport (v1.34.0) (33). Low-count genes were filtered prior to differential expression analysis with DESeq2 v1.50.2 (34) under a paired experimental design comparing three samples on the following groups: 1) conventionally expanded allo-iTreg vs Vitamin C-treated expanded allo-iTreg, 2) conventionally expanded allo-iTreg vs conventionally expanded allo-iTreg with cytokines, 3) Vitamin C-treated expanded allo-iTreg vs Vitamin C-treated expanded allo-iTregs with cytokines. Genes were classified as differentially expressed based on an absolute log_2_ fold change ≥ 1 and a false discovery rate (FDR) < 0.05.

### 2.10 Statistical analysis

Statistical analyses were conducted using Prism 8.0 software (GraphPad Software, San Diego, CA). Results are reported as mean ± SEM. The Shapiro–Wilk test was used to assess the normality of data distribution for each experimental condition. Since the data were normally distributed, a two-way analysis of variance (ANOVA) was performed, followed by Tukey’s and Sidak’s *post hoc* multiple-comparison tests. Statistical significance was defined as a p-value ≤ 0.05.

## 3 Results

### 3.1 Alloantigen-specific induced regulatory T cells expanded in the presence of Vitamin C display a CD25+ FOXP3+ stable phenotype

**Figure 1:**
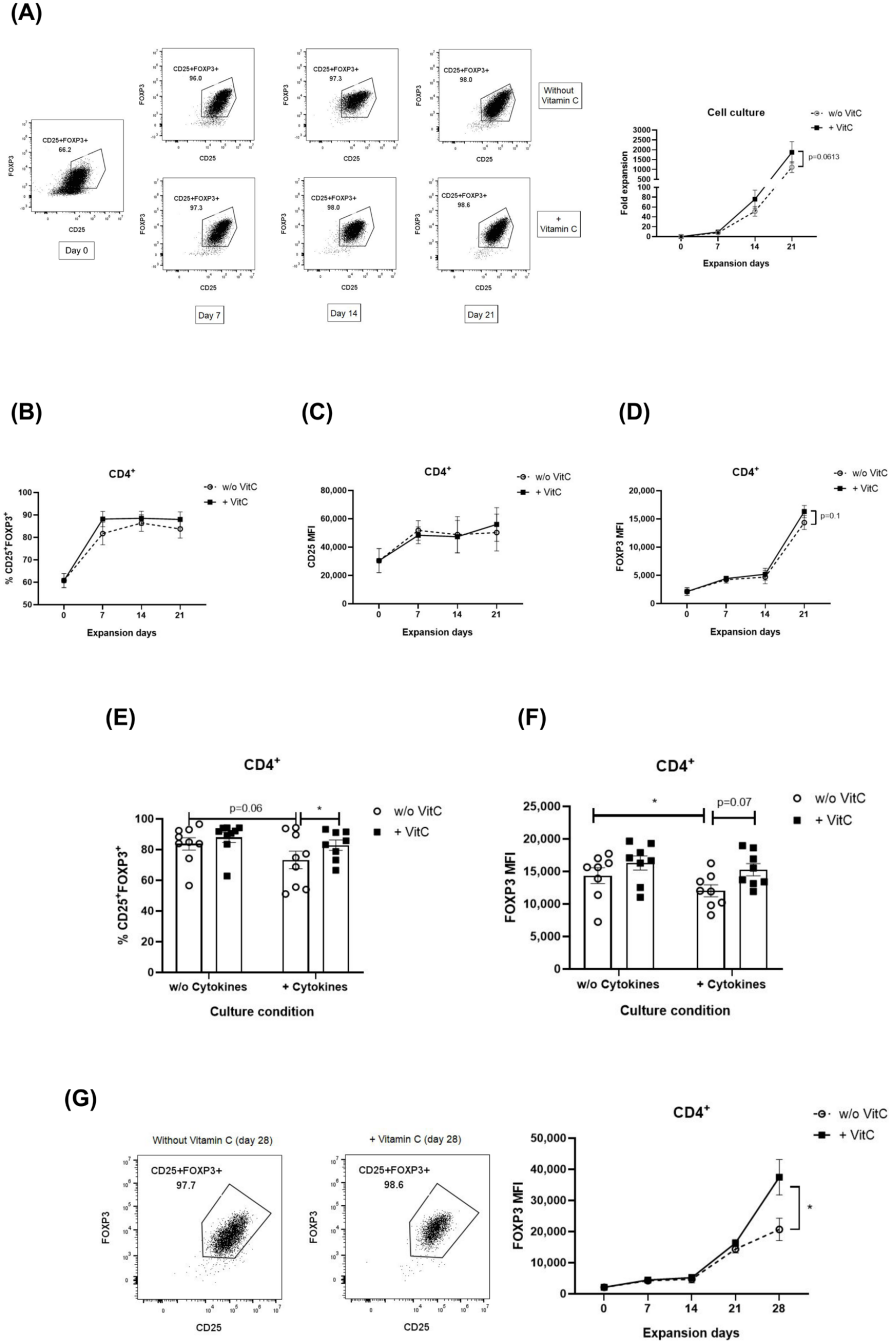
Tregs expanded with Vitamin C exhibit increased FOXP3 expression and enhanced stability of the CD25+FOXP3+ population when exposed to pro-inflammatory cytokines. Proliferating CD4+ CD25hi cells (allospecific iTregs) were purified from allogeneic co-cultures of naive T cells and mo-DC cells. These cells were polyclonally expanded for three weeks with or without Vitamin C. **(A)** Representative dot plots of CD4+CD25+FOXP3+ allo-iTreg population expanded for 7, 14 and 21 days. The right graph shows the fold expansion of iTregs over the three weeks of expansion. **(B)** Percentage of CD25+ FOXP3+ cells, **(C)** Mean fluorescence intensity (MFI) for CD25, and for FOXP3 **(D)** within the “gated” live CD4+ population. In the second week, IL-1, TNFα, and IL-6 were added into the culture for a week to assess stability under inflammatory conditions. The graphs display the percentage of CD25+ FOXP3+ cells **(E)** and FOXP3 MFI **(F)** within the live CD4+ population, measured on day 21. **(G)** Mean fluorescence intensity (MFI) of FOXP3 was measured on days 0, 14, 21, and 28 and showed a progressive, significant increase with Vitamin C supplementation. Representative dot plots of CD25+FOXP3+ allo-iTreg population on day 28 of expansion, are shown to the left of the graph. Data represent mean ± SEM from n=6–9 donors across three independent experiments. A two-way ANOVA and Sidak test were used for statistical analysis. P < 0.05 was considered statistically significant. * p < 0.05.

To evaluate the impact of Vitamin C supplementation on the *ex vivo* expansion of alloantigen-specific induced regulatory T cells (Allo-iTregs), cells were cultured for 3 weeks. After this period of expansion, total cell numbers, phenotypic characterization, and functional assays were performed. As shown in **Figure 1A**, allo-iTregs proliferated progressively throughout the culture period, with a trend toward a higher expansion rate in cultures supplemented with Vitamin C than in control cultures (day 21, 1873 ± 540.0 vs. 1118 ± 273.5, p=0.0613). The frequency of cells co-expressing CD25 and FOXP3 was higher from the first week of expansion in Vitamin C–supplemented cultures, with frequencies exceeding 85% by week 3 (**Figure 1B**). iTregs from both conditions maintained stable expression of CD25 and FOXP3 during the expansion period. FOXP3 expression levels at week 3 tended to be higher in cultures supplemented with Vitamin C compared to those without supplementation (16307.2 ± 1081.8 vs. 14366 ± 1233.7, p=0.1) (**Figures 1C, D**). After the second week of expansion, pro-inflammatory cytokines were added, and culture was extended for an additional week. Cultures supplemented with Vitamin C demonstrated a stability in retaining the CD25^+^FOXP3^+^ phenotype (88 ± 3.3 vs 83.9 ± 3.4) and sustained FOXP3 expression (16307.2 ± 1081.8 vs 15280.5 ± 940.3). In contrast, cultures that lacked Vitamin C showed a reduction in the percentage of CD25^+^FOXP3^+^ and the MFI of FOXP3, as shown in **Figure 1E** (83.8 ± 4.0 vs 73.3 ± 5.7, p=0.06) and **Figure 1F** (14366 ± 1233.7 vs 12026.6 ± 918.6, p=0.02). Interestingly, in some experiments when iTregs were expanded for 28 days, a significant increase in FOXP3 expression was observed in the presence of Vitamin C compared to untreated iTregs (37462.5 ± 5697 for Vitamin C–treated iTregs vs 20673.2 ± 3589, p=0.0258, day 28) (**Figure 1G**). These findings demonstrate that Vitamin C contributes to the preservation of phenotypic stability under pro-inflammatory conditions.

### 3.2 Allo-iTregs expanded with Vitamin C maintain the expression of co-inhibitory markers under pro-inflammatory conditions

In the third week of allo-iTreg expansion, the expression of key markers associated with their suppressive activity was evaluated, as well as their stability in inflammatory microenvironments, such as those found in allogeneic transplantation. To this end, cells were expanded with or without Vitamin C and subjected to simulated pro-inflammatory conditions by adding cytokines such as IL-1β, IL-6, and TNF-α.

**Figure 2:**
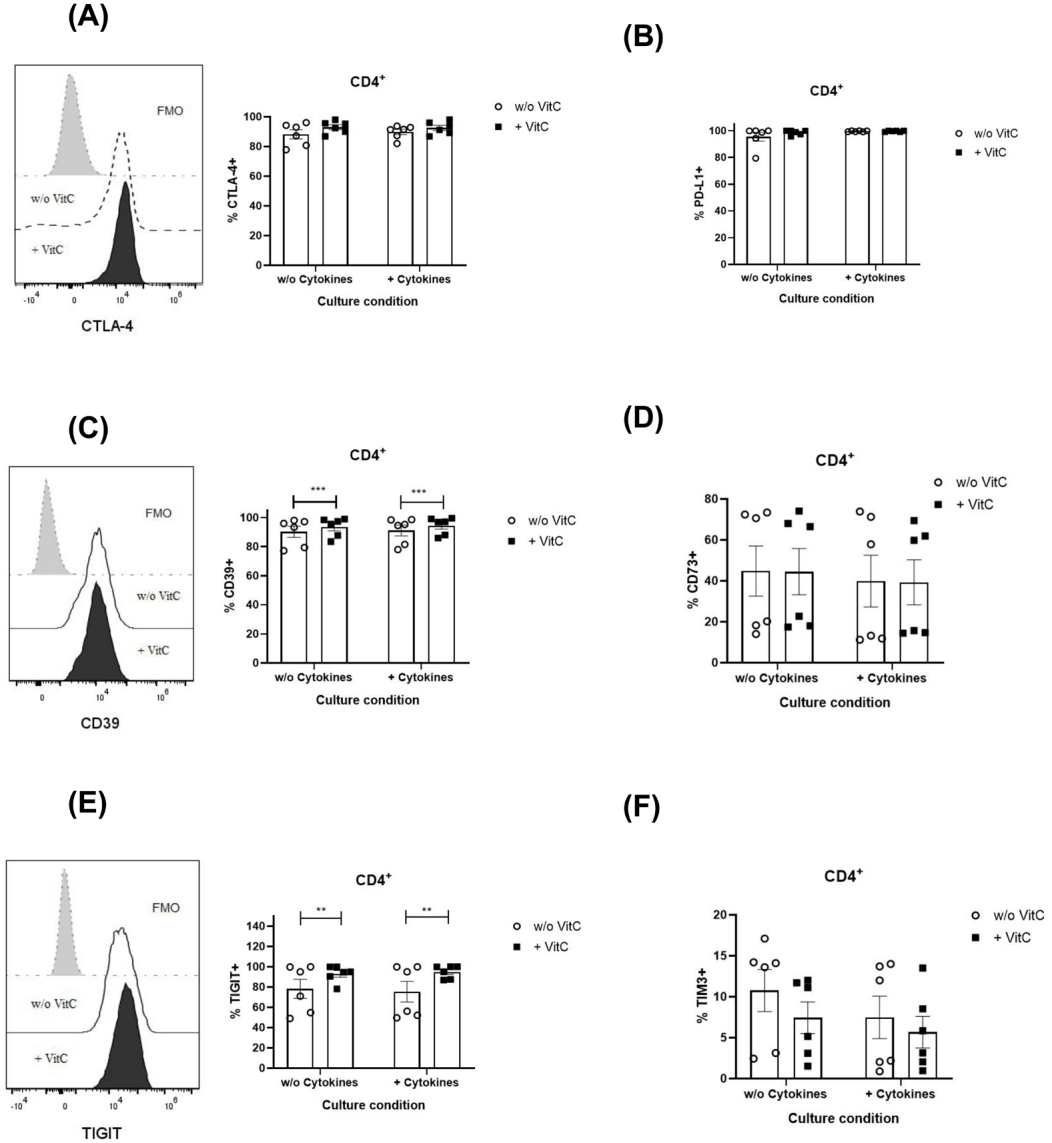
Allo-iTregs expanded in the presence of Vitamin C retain expression of suppressor immunophenotype markers following exposure to inflammatory cytokines. During the second week, IL-6, TNF-α, and IL-1β were added to the allo-iTreg cultures, and the expansion process continued for one more week. On day 21, expression of CTLA-4 **(A)**, PD-L1 **(B)**, CD39 **(C)**, CD73 **(D)**, TIGIT **(E)**, and TIM-3 **(F)** was assessed within the “gated” live CD4+ population, by flow cytometry. The frequency of each marker is presented. **(A–C, E)** Allo-iTregs express CTLA-4, PD-L1, CD39, and TIGIT at levels exceeding 70%, with higher expression observed in the presence of Vitamin C Representative histograms of the evaluated markers are shown to the left of each graph. Data represent mean ± SEM from n=6–9 donors across three independent experiments. A two-way ANOVA and Tukey test were used for statistical analysis. P < 0.05 was considered statistically significant. ** p < 0.01, and *** p < 0.001.

More than 90% of cells expressed CTLA-4 and PD-L1 in both culture conditions (without/with VitC), with no significant differences noted between groups (**Figures 2A, B**). Similarly, CD39 expression exceeded 90% in all conditions and appeared significantly higher in cultures treated with Vitamin C (93.5 ± 2.6 vs 90.2 ± 3.8, p=0.0008) (**Figure 2C**). Cultures treated with Vitamin C also displayed significantly higher TIGIT expression (93.03 ± 3.3 vs 78.3 ± 9.4, p=0.0032) (**Figure 2E**). CD73 expression varied substantially across independent experiments, but mean frequencies remained below 50% and did not differ significantly between conditions (**Figure 2D**). The exhaustion marker TIM-3 was expressed below 10% across all culture conditions and, again, differences between groups were not significant (**Figure 2F**). The addition of pro-inflammatory cytokines, under both conditions (with and without Vitamin C), did not alter the expression levels of the evaluated markers. These results support the phenotypic stability of our cells, not only in the expression of markers specific to their identity (CD25 and FOXP3), but also markers associated with their suppressor activity. If the markers are evaluated inside the CD25+FOXP3+ gating, the phenotypic frequencies and statistical significance trends remain nearly the same as evaluated inside the CD4+ gating (**Supplementary Figure 3**). This similarity is attributable to the high purity of this population within CD4+ cells (approximately 80%) and supports the robustness of the protocol for maintaining the identity of *de novo-generated*/expanded allo iTregs.

### 3.3 Allo-iTregs expanded in the presence of Vitamin C express chemokine receptors involved in migration to the allograft

To determine whether allo-iTregs acquire migratory properties toward peripheral tissues and secondary lymphoid organs, the expression of chemokine receptors associated with iTreg migration (CCR4, CCR5, CXCR3, and CCR7) was analyzed. The evaluation was conducted under culture conditions with or without Vitamin C supplementation and in the presence or absence of pro-inflammatory cytokines (IL-6, TNF-α, and IL-1β) to assess the stability of chemokine receptor expression in inflammatory microenvironments.

**Figure 3:**
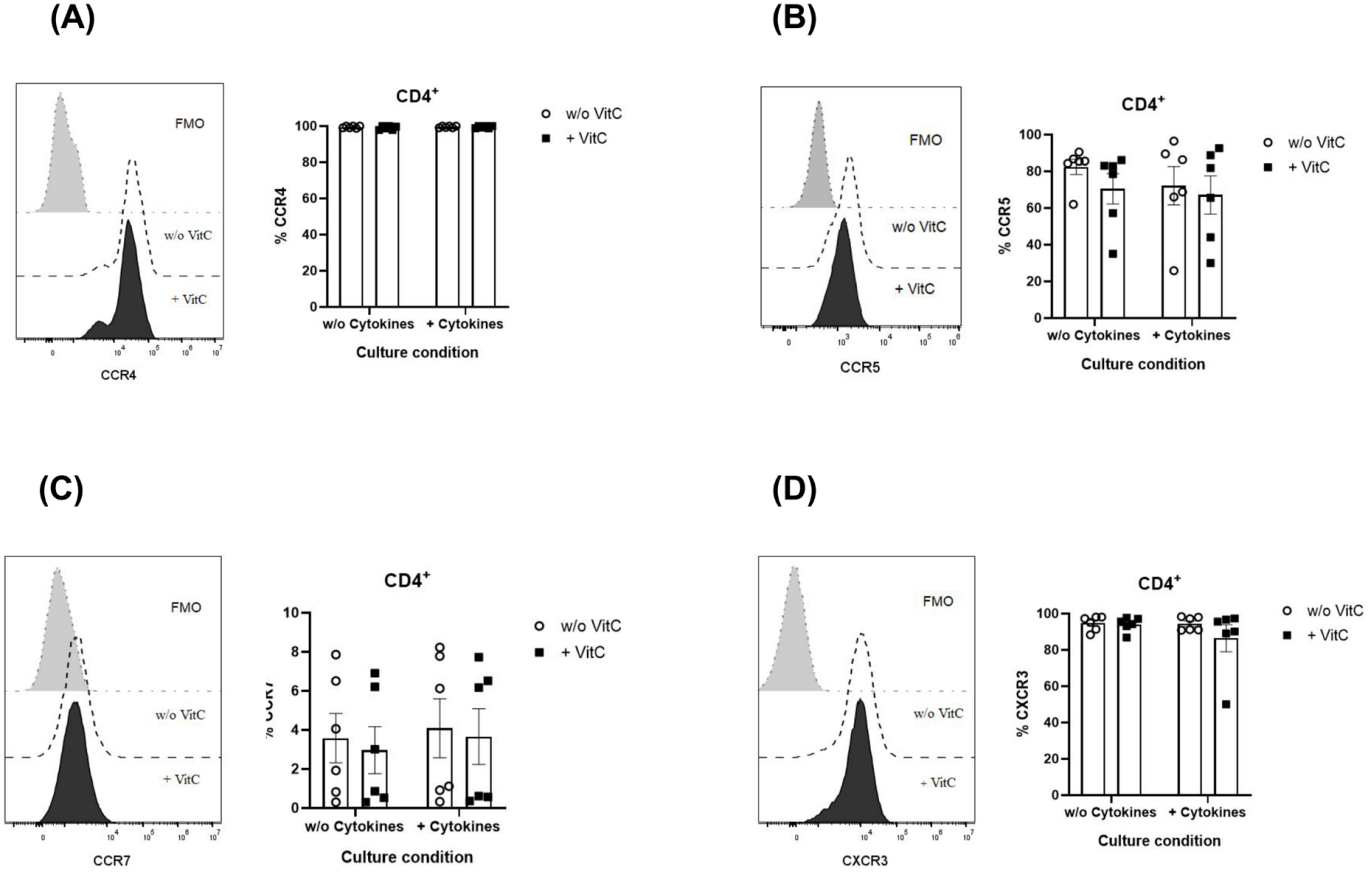
Allo-iTregs express chemokine receptors related to migration to the allograft, which are maintained in the presence of inflammatory cytokines. During the second week, IL-6, TNF-α, and IL-1β were added to the allo-iTreg cultures, and the expansion process continued for one more week On day 21, chemokine receptors related to migration were evaluated. The frequencies of CCR4 **(A)**, CCR5 **(B)**, CCR7 **(C)**, and CXCR3 **(D)** within the “gated” live CD4^+^ population, are shown. Expanded iTregs show high CCR4 expression and over 50% expression of CCR5 and CXCR3. CCR7 expression is low under all conditions. No significant differences were observed between the evaluated conditions. Representative histograms of the evaluated markers are shown to the left of each graph. Data represent mean ± SEM from n=6–9 donors across three independent experiments. A two-way ANOVA and Tukey test were used for statistical analysis. P < 0.05 was considered statistically significant.

Interestingly, the frequency of CCR4-expressing iTreg cells in cell cultures was close to 100% across all conditions evaluated (**Figure 3A**), with no difference between groups or effect of proinflammatory cytokines. Similarly, CXCR3 expression was greater than 80% (**Figure 3D**), although CCR5 expression was close to 70% across all conditions evaluated (**Figure 3B**). Interestingly, the frequency of CCR7-positive cells was less than 10% (**Figure 3C**). Under all conditions evaluated, no negative impact of added cytokines was observed. Therefore, in addition to remaining stable in an inflammatory microenvironment, allo-iTregs show a chemokine receptor profile with high expression of CCR4, CXCR3, and CCR5, but not CCR7. This pattern is commonly linked to homing to peripheral tissues and suggests that these cells may migrate toward the allograft.

### 3.4 Allo-iTregs expanded in the presence of Vitamin C and pro-inflammatory cytokines do not acquire an inflammatory IFN-γ (Th1) or IL-17 (Th17) profile

To determine whether allo-iTregs expanded for 3 weeks and exposed to pro-inflammatory conditions acquire an effector inflammatory phenotype that could indicate cellular instability, cytokine secretion assays were performed to assess the production of IFN-γ and IL-17A, which are hallmark cytokines of Th1 and Th17 cell subsets, respectively. Activated conventional T cells stimulated with anti-CD3/CD28 beads were used as a positive control.

**Figure 4:**
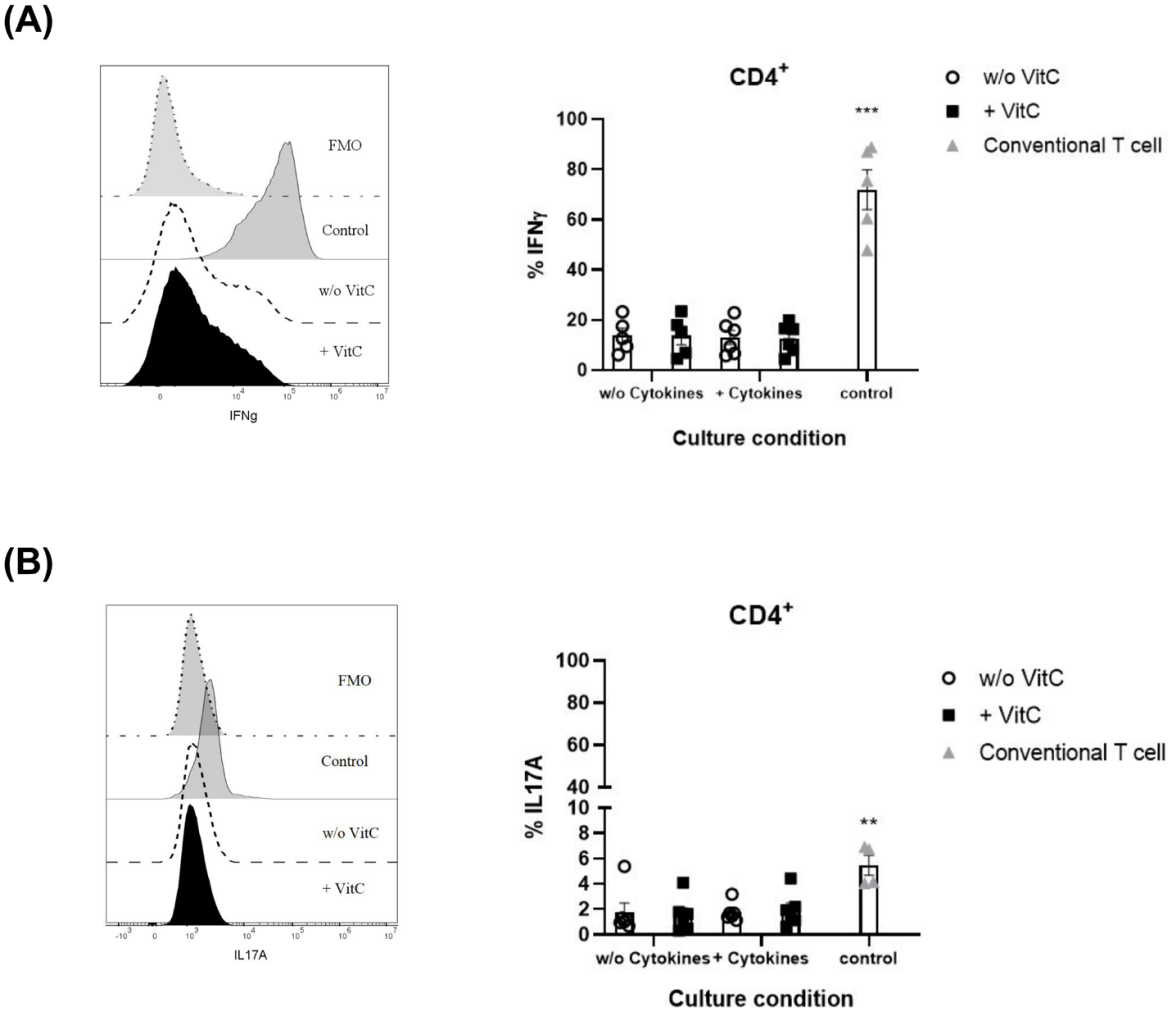
Expanded allo-iTregs do not develop a pro-inflammatory profile characterized by IFN-γ (Th1) or IL-17 (Th17) production. During the second week of expansion, IL-6, TNF-α, and IL-1β were added to the cultures for an additional week to assess phenotypic stability in a proinflammatory microenvironment by quantifying IFN-γ and IL-17 secretion. **(A)** IFN-γ production by expanded iTregs, with or without Vitamin C, remains significantly lower than that of conventional T cells. **(B)** IL-17A production by expanded iTregs, with or without Vitamin C, is also significantly lower than that of conventional T cells. No significant differences were observed between the evaluated conditions. The histograms display FMO and conventional T cell controls, as well as a representative sample of iTregs with and without Vitamin C Data represent mean ± SEM from n=6–9 donors across three independent experiments. A two-way ANOVA and Tukey test were used for statistical analysis. P < 0.05 was considered statistically significant. ** p < 0.01, and *** p < 0.001.

As shown in **Figure 4A**, allo-iTregs expanded in the presence or absence of Vitamin C exhibited low levels of IFN-γ production (<15% vs 71.9, p<0.0001)), which were significantly lower than those observed in conventional T cells. Likewise, IL-17A production by allo-iTregs expanded with or without Vitamin C was markedly reduced compared to conventional T cells (<2% vs. 5.46%, p=0.006)) (**Figure 4B**). The levels of both IFN-γ (13.7 ± 3.5 vs 12.6 ± 2.4, in condition with VitC) and IL-17A (1.5 ± 0.6 vs 1.9 ± 0.5, in condition with VitC) were not affected when allo-iTregs were expanded under pro-inflammatory conditions, further supporting the phenotypic stability of these cells.

### 3.5 Allo-iTregs expanded with Vitamin C and pro-inflammatory cytokines suppress conventional T cell proliferation

To evaluate the alloantigen-specific suppressive function of allo-iTregs against conventional T cells (CD3^+^CD4^+^ and CD3^+^CD8^+^), and to determine whether this function is maintained under inflammatory conditions, allogeneic coculture assays were performed.

**Figure 5:**
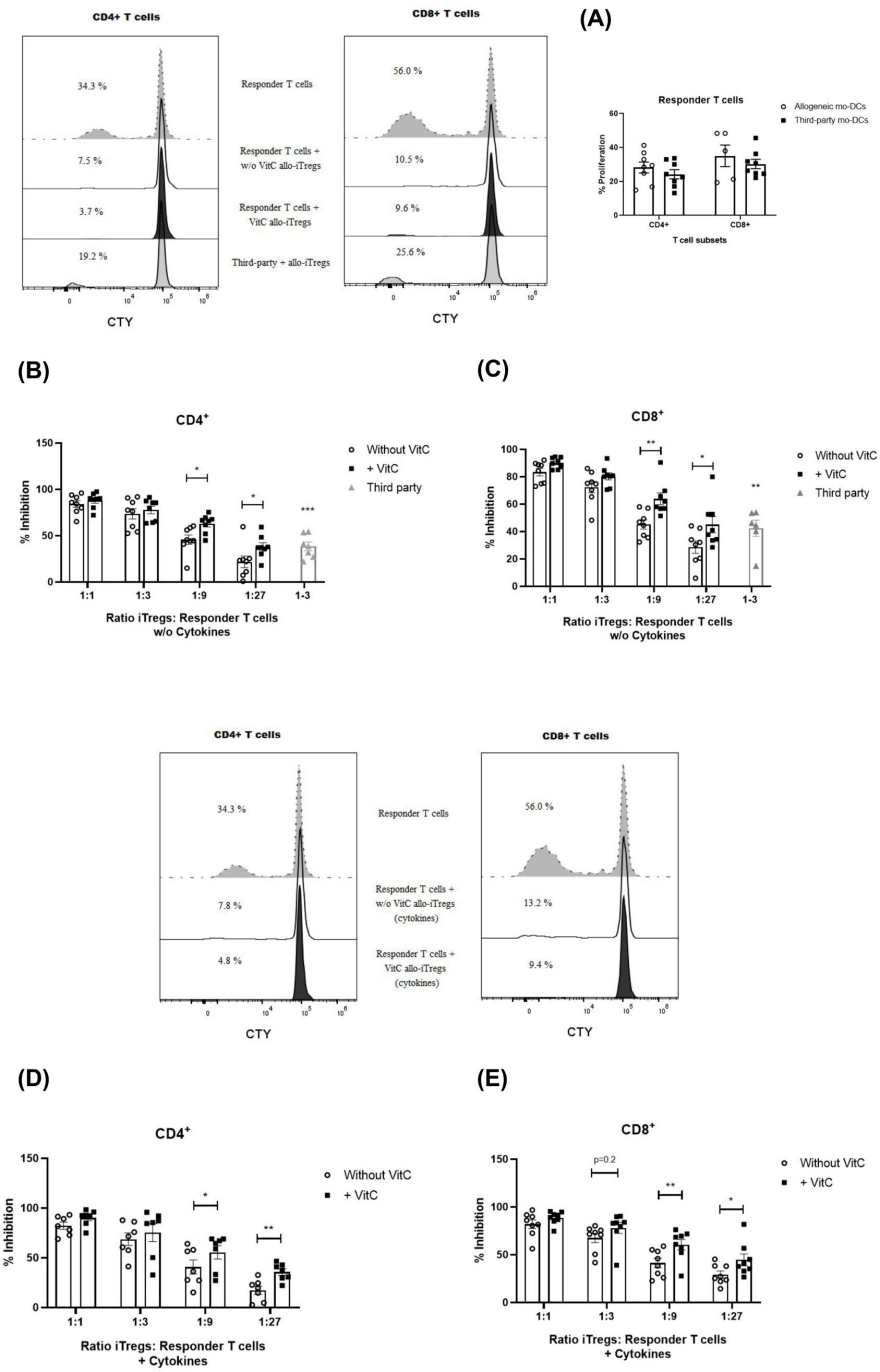
Expanded allospecific iTreg cells treated with Vitamin C enhance their suppressive function even after being activated in a pro-inflammatory environment. **(A)** Percentage of proliferation of responder T cells stimulated with allogeneic DCs vs. third party - DCs. On day 21 of expansion, the functional activity of induced regulatory T cells (iTregs) was evaluated by measuring their allo-specific suppression of conventional CD4+ T cell proliferation in the absence **(B)** or presence **(C)** of pro-inflammatory cytokines. In parallel, the inhibition of conventional CD8+ T cell proliferation was assessed when co-cultured with allo-iTregs under the same conditions **(D, E)**. Allo-iTregs treated with Vitamin C demonstrated greater suppression of both CD4+ and CD8+ T cell subpopulations, regardless of cytokine presence (white circle symbols), indicating enhanced suppressive capacity and functional stability. This suppression was antigen-specific, as it was more pronounced in co-cultures stimulated with allogeneic dendritic cells (DCs) compared to DCs from an unrelated donor (gray symbols, 1:3 ratio). Suppression was quantified as relative inhibition. Histograms show the proliferation of CD3^+^ T cells in the absence (responder T cells) or the presence of the allospecific expanded iTregs. The numbers indicate the percentage of proliferation, when iTregs:responder T cells are put into the proportion of 1:3. Data represent mean ± SEM from n=6–9 donors across three independent experiments. A two-way ANOVA and Tukey test were used for statistical analysis. P < 0.05 was considered statistically significant. * p < 0.05, ** p < 0.01, and *** p < 0.001.

Effector T cells stimulated only with mo-DCs showed robust and comparable basal proliferation in both allogeneic (28.52 ± 3.2 for CD4^+^ and 35.1 ± 6.3 for CD8^+^) and third-party (24.3 ± 2.7 for CD4^+^ and 30.3 ± 2.7 for CD8^+^) conditions (**Figure 5A**). These findings confirm that the system functioned as expected and that the reduced proliferation observed in co-cultures was due to iTreg-mediated suppression.

Interestingly, suppression of effector T cell proliferation was superior in co-cultures with Vitamin C- allo iTregs. This effect was significant at a 1:9 (62.9 ± 3.5 vs 45.7 ± 5.1, p=0.02) and 1:27 (38.0 ± 4.3 vs 21.8 ± 6.3, p=0.03) iTreg-to-effector T cell ratio for CD3^+^CD4^+^ T cells (**Figure 5B**), and at 1:9 (63.9 ± 4.3 vs 45.2 ± 3.4, p=0.004), and 1:27 (45.0 ± 5.9 vs 28.6 ± 4.5, p=0.02) ratios for CD3^+^CD8^+^ T cells (**Figure 5C**). Moreover, this difference persists following the addition of pro-inflammatory cytokines to the culture medium. The stability and enhanced suppressive capacity of VitC-expanded iTregs are evidenced by significant inhibition of proliferation at 1:9 and 1:27 ratios for CD3^+^CD4^+^ T cells (**Figure 5D**) and CD3^+^CD8^+^ T cells (**Figure 5E**) relative to the control.

Finally, cocultures stimulated with allogeneic mo-DCs exhibited greater suppression of CD4^+^ (73.7 ± 5.7 vs 38.4 ± 4.7, p=0.0004) and CD8^+^ (72.5 ± 4.2 vs 42.3 ± 5.9, p=0.001) T cell populations at 1:3 ratio, compared to those stimulated with third-party mo-DCs (gray bar). These findings suggest that iTreg-mediated suppression is antigen-specific and primarily directed toward the alloantigen used during their generation, rather than third-party antigens.

### 3.6 High-dimensional single-cell analysis supports a modulatory role of Vitamin C in iTreg biology

**Figure 6:**
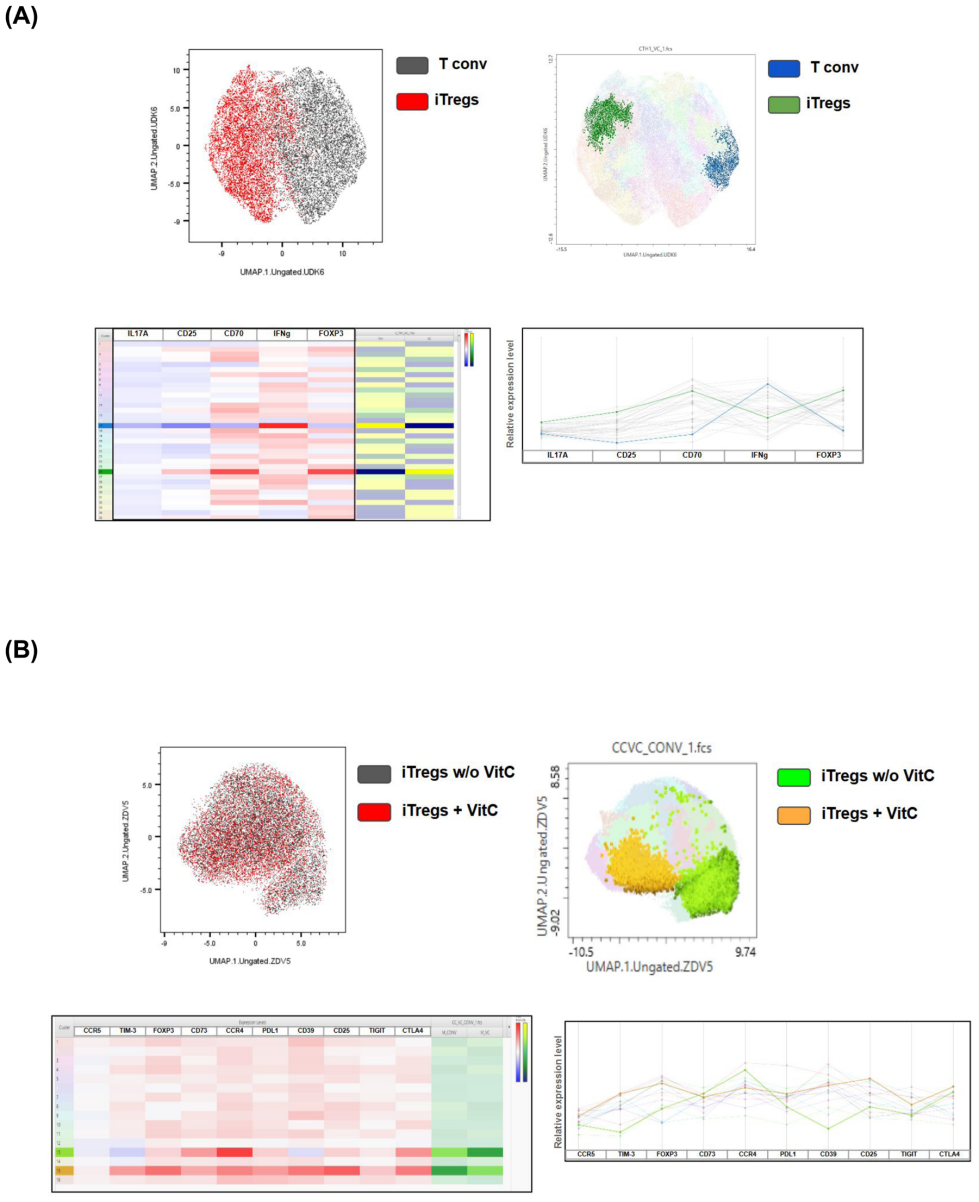
High-dimensional single-cell analysis supports a modulatory role of Vitamin C in iTreg biology. After 21 days of iTreg expansion with or without Vitamin C, high-dimensional analysis was conducted to assess marker expression profiles. **(A)** UMAP analysis compared live CD4^+^ iTregs (n=3, red) and conventional T cells (n=3, black), revealing distinct phenotypic profiles. Phenograph identified 35 clusters, and a heat map of median fluorescence expression levels was generated. Clusters 17 and 26 were selected to represent each population, with high FOXP3 expression observed in iTregs (green). **(B)** UMAP analysis of iTregs with (n=3) and without Vitamin C (n=3) showed phenotypic similarity between conditions. Of the 16 clusters generated by Phenograph, clusters 13 and 15 were compared, demonstrating that iTregs expanded with Vitamin C express high levels of TIM-3, FOXP3, CD39, and CD25 (orange).

To corroborate findings from conventional flow cytometry, high-dimensional flow cytometry was used to compare allo-iTreg cultures under different conditions. First, dimensionality reduction analysis (UMAP) on both allo-iTregs and conventional T cells (non-Tregs) revealed two main islands, one for each population, showing distinct phenotypic profiles. Several clusters on each island were exclusive to each population. Clusters corresponding to iTregs showed high FOXP3 expression, whereas those corresponding to conventional T cells showed low FOXP3 expression. Also, analysis of representative clusters showed that allo-iTregs expressed higher levels of CD25, CD70, and FOXP3 than conventional T cells (**Figure 6A**).

A direct comparison of allo-iTreg cultures with and without Vitamin C supplementation was subsequently conducted. UMAP analysis did not reveal distinct separation between these conditions, suggesting a high degree of overall phenotypic similarity between Vitamin C–treated and untreated iTregs. In accordance, Phenograph clustering did not produce condition-specific clusters, as each cluster contained cells from both culture conditions. Nevertheless, clusters with differential representation between conditions were identified. Specifically, comparing cluster 13 (without Vitamin C) and cluster 15 (with Vitamin C) showed that allo-iTregs expanded in the presence of Vitamin C expressed higher levels of TIM-3, FOXP3, CD39, CD25, and TIGIT. Furthermore, compared to cluster 13, cluster 15 shows a higher expression for PD-L1, TIGIT, and CTLA-4, markers associated with its suppressor function. Collectively, these findings indicate that although Vitamin C does not induce the emergence of phenotypically distinct iTreg populations, it exerts a relevant modulatory effect on their phenotype and potential functional properties (**Figure 6B**).

### 3.7 Decreased *FOXP3* TSDR methylation in long-term expanded allo-iTregs treated with Vitamin C.

**Figure 7:**
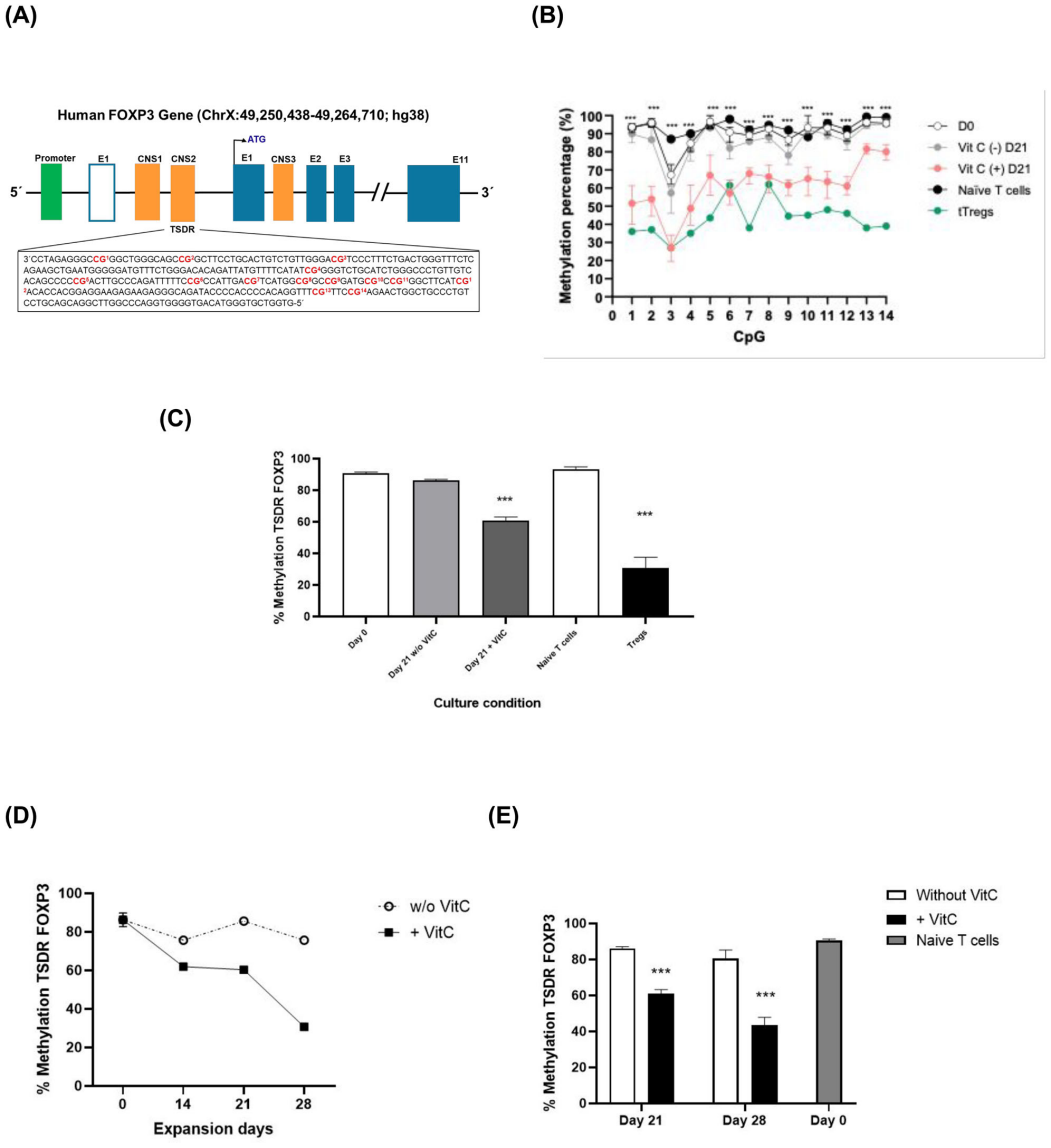
Vitamin C promotes demethylation of the TSDR region of FOXP3. **(A)** Schematic representation of the human FOXP*3* genomic locus showing Treg-specific demethylated region (TSDR) and the locations of the CpG dinucleotides (CG^1^–CG^14^) sequenced. CNS1–CNS3: conserved noncoding sequences 1-3. **(B)** Methylation percentages. of each 14 CpG sites, *** p < 0.001. **(C–E)** Percentage of the total methylation within the FOXP3 TSDR (considering 14 CpG sequences) at 21 **(C)** and 28 days **(E)** of expansion, respectively. Data represent mean of 3 experiments **(C)** (n=9) and 2 (n=6) **(E)** independent experiments. *** p < 0.001. **(D)** Kinetic analysis of TSDR methylation in a single donor across days 0, 14, 21, and 28 shows gradual demethylation of the FOXP3 locus. Statistical significance was assessed by two-way ANOVA followed by Tukey’s test. P < 0.05 was considered statistically significant.

Because demethylation of the *FOXP3* TSDR is a hallmark of stable regulatory T cell lineage commitment, the methylation status of this region was evaluated in allo-iTregs after three weeks of expansion. Pyrosequencing analysis demonstrated a significant reduction in the global percentage of methylated CpG sites in Vitamin C–treated iTregs (60.9 ± 2.4) compared with untreated iTregs (86.2 ± 0.9) and naïve T cells (93.4 ± 1.7). Thymic Tregs showed a methylation percentage of 30.9 ± 6.9 (**Figure 7**). Kinetic analysis of FOXP3 expression and TSDR demethylation at representative samples showed that TSDR demethylation is a progressive, cell division-dependent process, in which continuous Vitamin C supplementation drives a gradual and cumulative loss of TSDR methylation (43.5 ± 4.4 at day 28) compared with untreated iTregs (80.5 ± 4.8) (**Figures 7D, E**), which correlated with the increased FOXP3 expression described in **Figure 1G**.

### 3.8 Vitamin C modulates the transcriptional program of long-term expanded allo-iTregs

**Figure 8:**
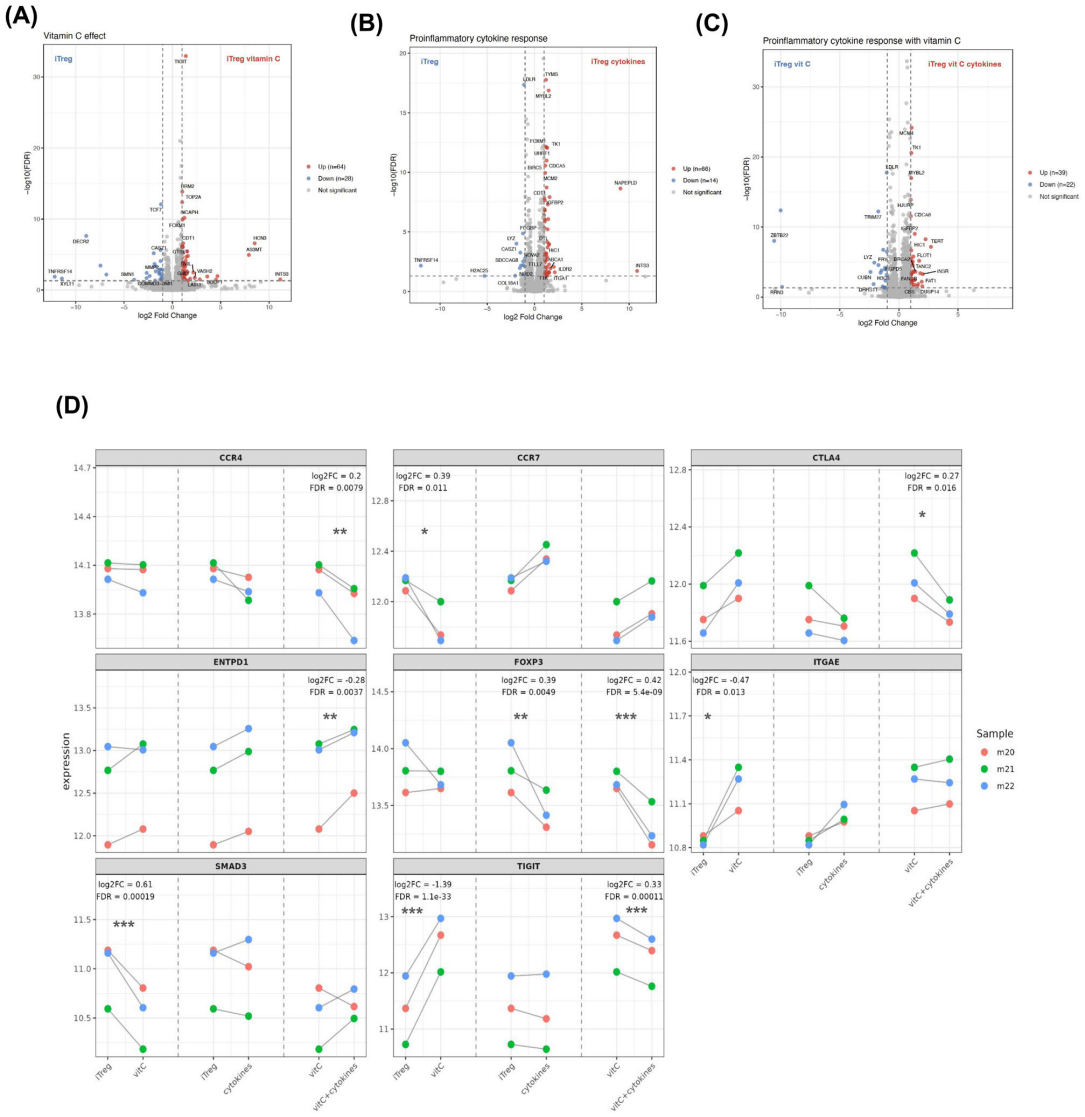
Transcriptional analysis of alloiTregs treated or not with Vitamin C in response to proinflammatory cytokines. **(A)** Effect of Vitamin C on immune regulation genes in allo-iTregs. **(B)** Effect of proinflammatory cytokines on untreated allo-iTregs. **(C)** Effect of proinflammatory cytokines on Vitamin C-treated allo iTregs. **(D)** Trajectory graph on relevant genes for T-cells comparing three independent samples for each treatment; when P < 0.05 it is indicated in the panel with the log2FoldChange. * p < 0.05, ** p < 0.01, and *** p < 0.001.

To assess the effect of Vitamin C on the transcriptome of *in vitro*-expanded allo-iTregs, we compared gene expression in allo-iTreg subpopulations from three different individuals, in the presence or absence of proinflammatory cytokines. As shown in **Figure 8A**, volcano plots from conventional allo-iTregs showed that Vitamin C had little influence in the transcriptomic profile of iTregs related to Treg function. 64 genes were upregulated and 26 were downregulated, but among them, only a few immune related genes appeared significantly upregulated in Vitamin C treated iTregs. Vitamin C–treated iTregs showed higher expression of the inhibitory receptor TIGIT and genes involved in DNA replication and cell proliferation, such as TOP2A, RRM2, and FOXM1. iTregs lacking Vitamin C showed elevated TCF7 and metabolic genes, such as DECR2. These results suggest Vitamin C induces regulatory markers and activation-associated genes.

We next evaluated the effect of the inflammatory microenvironment on iTregs. Cytokine exposure strongly increased expression of genes involved in DNA synthesis and cell cycle progression, including TYMS, TK1, MYBL2, FOXM1, and MCM2 (**Figure 8B**). TNFRSF14, an immune regulatory receptor, decreased after cytokine treatment. We tested whether Vitamin C changed this response. Proliferation-associated genes like MCM4, TK1, and MYBL2 stayed upregulated, but Vitamin C–treated cultures had fewer or milder transcriptional changes (up=66 vs up=39; **Figure 8C**). These data suggest cytokines promote proliferation and metabolic programs in iTregs, while Vitamin C may reduce response intensity. In summary, volcano plots demonstrated that most of the pathways enriched in Vitamin C or cytokine treated allo-iTersg were related to proliferation and cell division but not T cell differentiation or immune response pathways.

We also examined expression of Treg profile genes across all conditions. Although these genes did not meet differential expression thresholds in volcano plots, key regulatory markers—FOXP3, IL2RA, CTLA4, IKZF2, and TIGIT—were consistently expressed in all iTreg cultures. Only modest differences in transcript abundance were seen between conditions (**Figure 8D**). For example, Vitamin C induced upregulation of ITGAE which encodes for the integrin CD103, which is implicated in tissue retention and the maintenance of immune tolerance at mucosal sites (35). In addition TIGIT, an inhibitory receptor whose expression is associated with Treg suppressive capacity, was also upregulated in Vitamin-C treated cells (36).

Cytokine treatment of allo-iTregs led to a decreased FOXP3 transcription, consistent with previous findings reported in *pTregs* and *in vitro* iTregs exposed to inflammation (37). This effect on FOXP3 was maintained in Vitamin C-treated cells, although the differences showed only a decrease of 0.39 and 0.49 fold change, respectively. Notably, the effect of cytokine exposure on allo-iTregs and Vitamin C treated allo-iTregs showed a significant increase in CD39, an ectoenzyme important for Treg-mediated suppression (38) (**Figure 8D**). Other genes appeared to be modulated to a lesser extent, as shown by the heatmap analysis of the differentially expressed genes between conventionally expanded allo-iTregs and Vitamin C-treated iTregs, with or without cytokine exposure (**Supplementary Figure 4****).**

## 4 Discussion

While adoptive transfer of Tregs is a promising immunotherapy for inducing allograft tolerance, its success relies on the functional and phenotypic stability of the infused cells. Consequently, using induced Tregs (iTregs) remains challenging, as their epigenetic instability compromises both therapeutic efficacy and safety (8).

Building on this rationale, the present study introduces substantial modifications to our previously established protocol for the induction and expansion of allospecific iTregs (allo-iTregs) (29), aiming to optimize both their phenotypic stability and therapeutic applicability. A key modification was the incorporation of Vitamin C during the expansion phase to promote a stable epigenetic profile. By acting as a critical cofactor for TET enzymes (39), Vitamin C facilitates active DNA demethylation at key regulatory regions, most notably the *FOXP3* Treg-specific demethylated region (TSDR) (39–41).

Additionally, shortening the expansion period from six to three weeks optimized allo-iTreg functionality (29). This timeframe enhances clinical feasibility by accelerating personalized cellular product generation, improving the logistical and economic feasibility of large-scale GMP manufacturing, where shorter culture times help reduce production costs (42) and minimizes the risk of cellular exhaustion or unwanted effector phenotypes associated with prolonged *ex vivo* expansion (43, 44). Importantly, allo-iTregs exhibited exponential growth through day 21 in both Vitamin C-treated and control cultures, confirming that Vitamin C does not compromise their proliferative capacity (**Figure 1A**). By week three, cell numbers increased approximately 2,000-fold. Scaling up this process requires only 500,000 iTregs, which can be obtained from 1.5 million naive T cells. After three weeks of culture, these iTregs can be expanded to one billion antigen-specific regulatory T cells—a clinically relevant number to induce effective suppression in a 70 kg patient (45).

The ~60% CD25+FOXP3+ purity observed at Day 0 of expansion likely reflects the typical heterogeneity of *de novo* human iTreg induction rather than a deficiency in initial sorting purity. At this early time point, FOXP3 expression is predominantly driven by transient transcriptional activation mediated by TGF-β and TCR signaling pathways, while the TSDR region remains largely methylated (46). A progressive increase in the proportion of CD25^+^FOXP3^+^ cells (**Figure 1B**) and FOXP3 expression (**Figure 1D**) was observed over time, which principally relies on TGF-β for the *de novo* induction of FOXP3, and on rapamycin, to selectively expand Tregs by inhibiting effector T cell outgrowth and stabilizing the regulatory phenotype (47). Although FOXP3 expression is similar in both conditions during the first few days of culture, the difference becomes significant only at later stages (Day 28) in VitC conditions (**Figure 1G**), coinciding with consolidation of TSDR demethylation (**Figure 7E**). This supports the premise that Vitamin C promotes greater phenotypic stability within the regulatory lineage, aligning with reports that ascorbic acid facilitates *FOXP3* TSDR demethylation and sustains stable FOXP3 expression (26, 39).

A critical obstacle in clinical Treg-based therapies is the loss of FOXP3 expression upon encountering the pro-inflammatory milieu typical of solid organ transplantation (48–50). Previous studies document that cytokines like TNF-α severely compromise Treg stability and regulatory phenotype (51). Furthermore, inflammatory networks driven by IL-6 and IL-1β destabilize the regulatory lineage; notably, IL-1β potently drives FOXP3 downregulation, actively promoting Treg conversion into Th17 effectors cells (52–54). Importantly, while inflammatory signals drastically reduce FOXP3 levels during activation, our assays demonstrate that allospecific iTregs expanded with Vitamin C bypass this critical limitation, maintaining robust FOXP3 expression despite activation in a pro-inflammatory environment (**Figure 1E, F**). This marked resistance to cytokine-induced destabilization indicates that Vitamin C promotes long-term phenotypic stability through epigenetic regulation, intrinsically protecting the core identity of Tregs (39).

To exert their robust immunomodulatory functions, regulatory T cells rely on a diverse range of contact-dependent and contact-independent mechanisms. Our phenotypic characterization reveals that *ex vivo* expanded allo-iTregs constitutively express high levels of essential coinhibitory molecules such as CTLA-4 and PD-L1 (**Figures 2A, B**), which are critical for dampening antigen-presenting cell activation and effector T cell responses (48, 55). Vitamin C supplementation significantly upregulated the expression of CD39 and TIGIT (**Figures 2C, E**). CD39 represents the rate-limiting enzyme in the generation of immunosuppressive adenosine from pro-inflammatory extracellular ATP, a pathway vital for Treg-mediated protection in allograft transplantation (56). Similarly, high TIGIT expression identifies a lineage-stable, highly suppressive Treg subset capable of specifically inhibiting Th1/Th17 inflammatory responses including allograft rejection (57, 58). Most importantly, this suppressive profile remained completely unaltered even when the allospecific iTregs were challenged with a simulated pro-inflammatory milieu (IL-1β, IL-6, and TNF-α). Taken together, these findings suggest that Vitamin C not only preserves the core transcriptional identity of iTregs but also potentiates an enhanced suppressive machinery that remains phenotypically stable under the inflammatory conditions typical of solid organ transplantation. Elevated TIM-3 expression is frequently observed in response to chronic stimuli or within inflammatory environments, including tumor-infiltrating T cells and chronic viral infections (59, 60). In Tregs, TIM-3 expression correlates with enhanced suppressor activity, but also facilitates an effector state and induces a metabolic shift toward glycolysis (61), characteristics that align with an exhausted phenotype, in part through increased PI3K/AKT/mTOR signaling (62, 63). The observed low TIM-3 expression in induced Tregs (iTregs) in this study may indicate a more regulatory, rather than effector or exhausted, phenotype (**Figure 2F**).

For adoptive Treg therapy to successfully prevent solid organ rejection, the infused cells must possess the ability to efficiently home to the site of inflammation. Our results demonstrate that *ex vivo* expanded allo-iTregs exhibit a highly specialized tissue-homing chemokine receptor profile (**Figure 3**). Specifically, these cells present robust expressions of CCR4 (~100%), CXCR3 (>80%), and CCR5 (~60%), while lacking CCR7 (<10%). The downregulation of CCR7—a receptor essential for migration toward secondary lymphoid organs—combined with the high expression of inflammatory chemokine receptors indicates a strong propensity of these cells to migrate directly to peripheral inflamed tissues, such as the allograft (64). Previous studies have established that CCR4 and CCR5 are critical for Treg recruitment to allografts, facilitating localized immune tolerance and prolonging graft survival (65, 66). Furthermore, the substantial expression of CXCR3 enables these Tregs to specifically traffic to sites of Th1-mediated inflammation, enabling them to co-localize with and potently suppress pathogenic alloreactive effector T cells within the graft (64). The concerted expression of these receptors is widely recognized as essential for maximizing the protective function of Tregs in transplantation settings (67). Crucially, the addition of a simulated pro-inflammatory milieu (IL-1β, IL-6, and TNF-α) did not alter this expression profile across any of the evaluated conditions. Since these findings are derived from surface immunophenotyping, subsequent *in vitro* chemotaxis assays and *in vivo* biodistribution studies are required to confirm the functional migratory capacity and localized suppressive activity of these cells.

Another major safety in clinical iTreg therapy is their potential reprogramming into pathogenic Th1 and Th17 cells upon exposure to inflammatory environments (54, 68). Such conversion could abrogate graft tolerance and actively exacerbates allograft rejection (69). Addressing this, our functional assays reveal that *ex vivo* expanded allo-iTregs, regardless of Vitamin C supplementation, maintain remarkably low IFN-γ and IL-17A production compared to activated conventional T cells (**Figure 4**). Notably, this restricted effector cytokine profile remained rigidly unaltered even under simulated pro-inflammatory conditions. This unresponsiveness confirms our overall protocol yields a highly stable regulatory product that resists acquiring a deleterious effector phenotype. It is worth noting that a fraction (10–15%) of IFN-γ-producing cells is present in the allo-iTreg populations. Current evidence suggests that this subpopulation does not necessarily reflect lineage instability—especially given the high FOXP3 expression and TSDR demethylation observed in our study—but rather that these are Tregs that adopt context-dependent functions. Findings reveal that IFN-γ production by Tregs is essential to mediate local immune responses to prevent tissue damage, as in graft-versus-host disease (GVHD) (70), and during inflammation, these cells adopt a Th1-like transcriptional program to migrate and suppress local Th1 responses (71), as supported by the high CXCR3 receptor expression observed in our study.

These findings must be contextualized with previous reports on Vitamin C’s pleiotropic effects. Song et al. showed that under Th17-polarizing conditions, Vitamin C enhances IL-17 expression by acting as a cofactor for *Jmjd2* demethylases, reducing suppressive H3K9me3 marks at the *Il17* locus (72). While this suggests a potential risk, our results demonstrate Vitamin C does not promote IL-17A secretion during allo-iTreg expansion, even under inflammatory challenges. This discrepancy highlights the context-dependent nature of Vitamin C-mediated epigenetic remodeling, which in our system is profoundly shaped by rapamycin. Rapamycin, a specific mTOR inhibitor, potently suppresses Th17 differentiation while promoting FOXP3^+^ Treg expansion (73). In our protocol, rapamycin could metabolically inhibit Th17 polarization, favoring Vitamin C-driven, TET-mediated *FOXP3* demethylation and resulting sustained FOXP3 expression, supporting the overall safety of this culture strategy.

The therapeutic utility of expanded allo-iTregs relies on their potent, antigen-specific suppressive capacity. Our coculture assays demonstrate that these cells effectively inhibit CD4^+^ and CD8^+^ effector T cell proliferation. Vitamin C supplementation enhanced this suppressive efficacy, particularly at highly iTreg-to-effector ratios (1:9; 1:27 for CD4^+^, and 1:9, 1:27 for CD8^+^ T cells) (**Figure 5B, C**), indicating superior per-cell functional potency. Furthermore, this suppression was antigen-specific; iTregs challenged with third-party dendritic cells showed reduced ability to suppress responder T cells, compared to those stimulated with the original alloantigen (gray symbols). A previous study by this group demonstrated that allo-iTregs generated using the same induction and expansion protocol consume IL-2 from the microenvironment and produce significant levels of IL-10 during their suppressive function (29). Given that the lineage identity and robust regulatory phenotype (high expression of CTLA-4, PD-L1, CD39, and CD73) remain consistent across the cultures in this study, we anticipate that the secretion of suppressive cytokines, such as IL-10, acts in profound synergy with these membrane mechanisms.

This robust, alloantigen-specific regulatory profile is critical for clinical translation, as strong *in vitro* antigen-specific suppression directly correlates with superior *in vivo* regulatory function and prolonged allograft survival, avoiding the risks of generalized immunosuppression (28). Beyond basal potency, the enhanced suppressive function of our expanded allo-iTregs was fully preserved despite exposure to a pro-inflammatory cytokine challenge (TNF-α, IL-6, and IL-1β) (**Figures 5D, E**). The ability to sustain robust suppression of effector subsets under these hostile conditions confirms their remarkable functional resilience. This finding aligns with evidence demonstrating that adequately stabilized Tregs can resist inflammatory abrogation, maintaining their specialized immunosuppressive mechanisms even during active tissue inflammation (26). Ultimately, integrating Vitamin C into our expansion protocol yields a highly potent, antigen-specific, and functionally stable allo-iTreg product.

High-dimensional analysis of multiparametric flow cytometry data demonstrated that the allo-iTreg population is distinguishable from conventional T cells, with both populations acquiring well-defined phenotypic profiles under the specified culture conditions (**Figure 6A**). These findings indicate that the developed protocol is efficient and consistent, yielding a population with an immunoregulatory identity clearly distinct from that of conventional effector T cells. Comparison of the allo-iTreg populations generated with or without Vitamin C did not reveal distinct separation (**Figure 6B**), indicating a high degree of phenotypic similarity and that Vitamin C supplementation does not induce abnormal subpopulations or alter the overall identity of allo-iTregs, supporting biosafety in cell therapies. Analysis of the most representative clusters in each condition demonstrated higher expression of molecules associated with identity, stability, and suppressor function in the Vitamin C representative cluster. This finding suggests that Vitamin C may potentiate characteristics associated with enhanced suppressor capacity and functional stability, which are important for ex vivo expansion strategies in therapeutic applications, where Treg stability and functional potency are critical determinants (74).

Pyrosequencing analysis revealed that Vitamin C supplementation during the long-term expansion of allo-iTregs leads to a significant reduction in DNA methylation across 14 CpG sites within the TSDR of the *FOXP3* locus (**Figure 7**). This result is of considerable importance, as stable demethylation of the *FOXP3* TSDR is widely recognized as a key epigenetic hallmark indicative of regulatory T cell lineage commitment and sustained functional stability (28, 75). It is worth mentioning that Vitamin C treatment of expanded allo-iTregs resulted in a strong demethylation effect during iTreg expansion, reaching a 50% reduction of the percentage of TSDR methylation (80% in untreated versus 40% in vitC-treated iTregs) at 28 days of expansion. Kinetic TSDR demethylation in a representative sample, shows that this process does not show an early peak; it progresses gradually with cell division, and continuous vitamin C supplementation drives a cumulative loss of TSDR methylation in the FOXP3 gene (**Figure 7D**). Therefore, our data showed that Vitamin C promotes an epigenetic profile in our expanded allo-iTregs that approaches that found in freshly isolated thymic Tregs (30%), and which correlates with their significant FOXP3 expression (**Figure 1G** and **7E**) (16). Demethylation of TSDR region of the *Foxp3* gene increases chromatin accessibility to transcription factors such as Ets-1, which specifically bind to the demethylated CpG islands and are integral for sustained transcriptional activity and secure FOXP3 expression within Tregs to the functional maintenance of the Treg lineage (18). Ets-1 is part of a protein complex that includes other transcription factors such as CREB/ATF and NF-κB that orchestrate the enhancer activity within the *FOXP3* TSDR. Disruption of these interactions impairs Treg stability and suppressive function, underscoring their epigenetic significance in Treg homeostasis (76). Consistent with these observations, our results show that cells expanded in the absence of Vitamin C retain high levels of TSDR methylation, suggesting reduced epigenetic stability of allo-iTregs during prolonged culture.

The effect of Vitamin C on TSDR demethylation can be mechanistically explained by its role as a cofactor for Fe²^+^/α-ketoglutarate–dependent dioxygenases, particularly the TET family of enzymes, which catalyze the oxidation of 5-methylcytosine and promote active DNA demethylation (75). Several studies have demonstrated that Vitamin C enhances TET activity and promotes epigenetic remodeling in multiple cellular contexts, including pluripotent stem cells and T lymphocytes (77). TET2 has been identified as an essential demethylation mediator of key regulatory regions, such as the TSDR element within the *FOXP3* locus, which is essential for the acquisition and maintenance of the functional identity of Treg cells within the thymic microenvironment. Additionally, the activity of other TET enzymes, such as TET3, has also been implicated in the early stages of thymic Treg differentiation, facilitating chromatin remodelling and promoting transcription of the *FOXP3* locus in response to TCR and IL-2 signalling, which is crucial for the efficient generation of functional Tregs in the thymus. This critical, non-redundant function is supported by evidence showing selective roles for TET3 in the generation of CD25^−^FOXP3^lo^ Treg precursors and TCR-dependent IL-2 production, highlighting a broader epigenetic pathway that fine-tunes Treg lineage commitment during thymic differentiation (78). Additionally, recent analyses demonstrate that while Vitamin C enhances TET enzymatic activity, the loss of individual TET paralogs, particularly TET2, cannot be fully compensated by Vitamin C supplementation, underscoring the non-redundant contributions of each TET protein to active DNA demethylation and Treg-specific epigenetic programming (79). These findings illuminate a complex regulatory network, where both Vitamin C availability and TET paralog integrity are essential for maintaining the epigenetic landscape associated with Treg lineage stability and immunosuppressive activity.

Although Vitamin C-treated allo-iTregs exhibited greater demethylation of *FOXP3* and other Treg-specific epigenetic signature genes, along with increased stability of *FOXP3* expression, RNA-seq analysis indicated that its impact is predominantly epigenetic rather than transcriptomic. Few genes were clearly modulated by Vitamin C at the transcriptional level, although the fold change was not sufficient to be identified in volcano plots. Interestingly, although vitamin C increased TSDR demethylation, we detected no significant changes in transcription of TET or DNMT genes between untreated and vitamin C-treated iTregs (data not shown). This is consistent with previous studies showing that vitamin C promotes TSDR demethylation by increasing TET enzymatic activity, as reflected by enhanced conversion of 5-methylcytosine to 5-hydroxymethylcytosine, without altering TET or DNMT transcription (21, 26, 27).

In contrast, there were more differences in gene expression both in untreated iTregs and Vitamin C-treated iTregs after exposure to a proinflammatory environment. However, Heatmap analysis showed that allo-iTregs not treated with Vitamin C underwent a more profound transcriptional alteration in genes related to T cell differentiation, whereas Vitamin C- treated allo iTregs, maintained a more stable profile (**Supplementary Figure 4**).

Translating these epigenetic benefits to human iTregs cultures, however, has proven challenging. Most of these studies examined polyclonal, short-term mouse iTreg cultures under non-inflammatory conditions, and support a role for vitamin C in generating potentially stable iTregs. In one study of human iTregs stimulated with IL-6, vitamin C treatment produced only 12% demethylation after 6 days of culture (80), suggesting that vitamin C is less effective at reducing methylation in human iTregs than in mouse iTregs, where Vit C-supplementation achieved demethylation levels comparable to those of tTregs. In contrast, our 28-day expansion protocol with continuous Vitamin C supplementation overcame demethylation resistance in the human TSDR, reaching 60% demethylation by week 3 and maintaining 40% through week 4. These findings suggest that human cells require prolonged, uninterrupted exposure to ascorbate to stabilize lineage commitment, unlike the more rapid epigenetic stabilization observed in murine models. In this context, our study demonstrates the feasibility of expanding antigen-specific human iTregs over the long term, while preserving phenotype and function in a proinflammatory microenvironment, which remains a concern for long-term tolerance induction in transplanted patients.

Future research should incorporate integrated epigenetic and functional analyses to rigorously evaluate the persistence and immunosuppressive capacity of Vitamin C-treated allo-iTregs in both preclinical and clinical settings. Furthermore, comprehensive validation and longitudinal stability assessments will be indispensable for establishing the translational potential of this approach in the development of regulatory T cell-based immunotherapies.

We conclude that, Vitamin C facilitates the maintenance of allospecific iTreg identity and function in challenging immune environments. The findings indicate that allospecific iTregs treated with Vitamin C may be especially effective for immunotherapy approaches focused on achieving lasting tolerance in transplant patients.

## Data Availability

The original contributions presented in the study are included in the article/**Supplementary Material**, further inquiries can be directed to the corresponding author/s.

## References

[1] Lee AY Jeong J Heo K-N Park S Ah Y-M Han JM . Complications associated with immunosuppressive agents in solid organ transplant recipients: A nationwide analysis. J Clin Med. (2025) 14:3602. doi: 10.3390/jcm14103602 40429597 PMC12112735

[2] Blein T Ayas N Charbonnier S Gil A Leon J Zuber J . Tolerance induction strategies in organ transplantation: Current status and future perspectives. Transpl Int. (2025) 38:14958. doi: 10.3389/ti.2025.14958 41127478 PMC12539449

[3] Mikami N Sakaguchi S . Regulatory T cells in autoimmune kidney diseases and transplantation. Nat Rev Nephrol. (2023) 19:544–57. doi: 10.1038/s41581-023-00733-w 37400628

[4] Zou C Li P Li B Sparwasser T Yuan J . Next steps in regulatory T cells: Biology and clinical application. Cell. (2026) 189:6–22. doi: 10.1016/j.cell.2025.11.035 41512846

[5] Sakaguchi S Mikami N Wing JB Tanaka A Ichiyama K Ohkura N . Regulatory T cells and human disease. Annu Rev Immunol. (2020) 38:541–66. doi: 10.1146/annurev-immunol-042718-041717 32017635

[6] Du Y Fang Q Zheng S-G . Regulatory T cells: Concept, classification, phenotype, and biological characteristics. Adv Exp Med Biol. (2021) 1278:1–31. doi: 10.1007/978-981-15-6407-9_1 33523440

[7] Kanamori M Nakatsukasa H Okada M Lu Q Yoshimura A . Induced regulatory T cells: Their development, stability, and applications. Trends Immunol. (2016) 37:803–11. doi: 10.1016/j.it.2016.08.012 27623114

[8] Alvarez-Salazar EK Cortés-Hernández A Arteaga-Cruz S Soldevila G . Induced regulatory T cells as immunotherapy in allotransplantation and autoimmunity: Challenges and opportunities. J Leukoc Biol. (2024) 116:947–65. doi: 10.1093/jleuko/qiae062 38630873

[9] Mukai M Takahashi H Kubo Y Asahina Y Iriki H Nomura H . Conversion of pathogenic T cells into functionally stabilized Treg cells for antigen-specific immunosuppression in pemphigus vulgaris. Sci Transl Med. (2025) 17. doi: 10.1126/scitranslmed.adq9913 41124284

[10] Maganto-García E Bu D-X Tarrio ML Alcaide P Newton G Griffin GK . Foxp3+-inducible regulatory T cells suppress endothelial activation and leukocyte recruitment. J Immunol. (2011) 187:3521–9. doi: 10.4049/jimmunol.1003947 21873519 PMC3217244

[11] Zheng SG Wang J Horwitz DA . Cutting edge: Foxp3+CD4+CD25+ regulatory T cells induced by IL-2 and TGF-beta are resistant to Th17 conversion by IL-6. J Immunol. (2008) 180:7112–6. doi: 10.4049/jimmunol.180.11.7112 18490709

[12] Luo Y Xue Y Wang J Dang J Fang Q Huang G . Negligible effect of sodium chloride on the development and function of TGF-β-induced CD4+ Foxp3+ regulatory T cells. Cell Rep. (2019) 26:1869–1879.e3. doi: 10.1016/j.celrep.2019.01.066 30759396 PMC6948355

[13] Nguyen T-L Makhlouf NT Anthony BA Teague RM DiPaolo RJ . *In vitro* induced regulatory T cells are unique from endogenous regulatory T cells and effective at suppressing late stages of ongoing autoimmunity. PloS One. (2014) 9:e104698. doi: 10.1371/journal.pone.0104698 25119105 PMC4131893

[14] Yang F Yanes A Li M Heizer P Linatoc I Stephens ME . Differential regulation of Treg stability in human naïve and effector Treg subsets by TGFβ-signaling via ARKADIA-SKI axis. Front Immunol. (2025) 16. doi: 10.3389/fimmu.2025.1636434 40995370 PMC12454061

[15] Xie W-W Huang J-B Zhou Y-C Yuan J-Y Feng J-X Shi X-H . The immunobiology and therapeutic potential of regulatory T cells in autoimmune diseases and allergic diseases. Front Immunol. (2026) 16. doi: 10.3389/fimmu.2025.1709915 41607801 PMC12835373

[16] Toker A Engelbert D Garg G Polansky JK Floess S Miyao T . Active demethylation of the Foxp3 locus leads to the generation of stable regulatory T cells within the thymus. J Immunol. (2013) 190:3180–8. doi: 10.4049/jimmunol.1203473 23420886

[17] Kitagawa Y Ohkura N Kidani Y Vandenbon A Hirota K Kawakami R . Guidance of regulatory T cell development by Satb1-dependent super-enhancer establishment. Nat Immunol. (2017) 18:173–83. doi: 10.1038/ni.3646 27992401 PMC5582804

[18] Polansky JK Schreiber L Thelemann C Ludwig L Krüger M Baumgrass R . Methylation matters: Binding of Ets-1 to the demethylated Foxp3 gene contributes to the stabilization of Foxp3 expression in regulatory T cells. J Mol Med. (2010) 88:1029–40. doi: 10.1007/s00109-010-0642-1 20574810 PMC2943068

[19] Candia E Reyes P Covian C Rodriguez F Wainstein N Morales J . Single and combined effect of retinoic acid and rapamycin modulate the generation, activity and homing potential of induced human regulatory T cells. PloS One. (2017) 12:e0182009. doi: 10.1371/journal.pone.0182009 28746369 PMC5529012

[20] Luu M Riester Z Baldrich A Reichardt N Yuille S Busetti A . Microbial short-chain fatty acids modulate CD8+ T cell responses and improve adoptive immunotherapy for cancer. Nat Commun. (2021) 12:4077. doi: 10.1038/s41467-021-24331-1 34210970 PMC8249424

[21] Yue X Trifari S Äijö T Tsagaratou A Pastor WA Zepeda-Martínez JA . Control of Foxp3 stability through modulation of TET activity. J Exp Med. (2016) 213:377–97. doi: 10.1084/jem.20151438 26903244 PMC4813667

[22] Akamatsu M Mikami N Ohkura N Kawakami R Kitagawa Y Sugimoto A . Conversion of antigen-specific effector/memory T cells into Foxp3-expressing Treg cells by inhibition of CDK8/19. Sci Immunol. (2019) 4:eaaw2707. doi: 10.1126/sciimmunol.aaw2707 31653719

[23] Mikami N Kawakami R Chen KY Sugimoto A Ohkura N Sakaguchi S . Epigenetic conversion of conventional T cells into regulatory T cells by CD28 signal deprivation. Proc Natl Acad Sci USA. (2020) 117:12258–68. doi: 10.1073/pnas.1922600117 32414925 PMC7275710

[24] Okada M Kanamori M Someya K Nakatsukasa H Yoshimura A . Stabilization of Foxp3 expression by CRISPR-dCas9-based epigenome editing in mouse primary T cells. Epigenet Chromatin. (2017) 10:24. doi: 10.1186/s13072-017-0129-1 28503202 PMC5422987

[25] Kressler C Gasparoni G Nordström K Hamo D Salhab A Dimitropoulos C . Targeted de-methylation of the FOXP3-TSDR is sufficient to induce physiological FOXP3 expression but not a functional Treg phenotype. Front Immunol. (2021) 11. doi: 10.3389/fimmu.2020.609891 33488615 PMC7817622

[26] Sasidharan Nair V Song MH Oh KI . Vitamin C facilitates demethylation of the Foxp3 enhancer in a Tet-dependent manner. J Immunol. (2016) 196:2119–31. doi: 10.4049/jimmunol.1502352 26826239

[27] Someya K Nakatsukasa H Ito M Kondo T Tateda K-I Akanuma T . Improvement of Foxp3 stability through CNS2 demethylation by TET enzyme induction and activation. Int Immunol. (2017) 29:365–75. doi: 10.1093/intimm/dxx049 29048538 PMC5890887

[28] Nikolouli E Hardtke-Wolenski M Hapke M Beckstette M Geffers R Floess S . Alloantigen-induced regulatory T cells generated in presence of vitamin C display enhanced stability of Foxp3 expression and promote skin allograft acceptance. Front Immunol. (2017) 8. doi: 10.3389/fimmu.2017.00748 28702031 PMC5487376

[29] Alvarez-Salazar EK Cortés-Hernández A Arteaga-Cruz S Alberú-Gómez J Soldevila G . Large-scale generation of human allospecific induced Tregs with functional stability for use in immunotherapy in transplantation. Front Immunol. (2020) 11:375. doi: 10.3389/fimmu.2020.00375 32300340 PMC7142244

[30] Ou K Hamo D Schulze A Roemhild A Kaiser D Gasparoni G . Strong expansion of human regulatory T cells for adoptive cell therapy results in epigenetic changes which may impact their survival and function. Front Cell Dev Biol. (2021) 9:751590. doi: 10.3389/fcell.2021.751590 34869339 PMC8639223

[31] Chen S Zhou Y Chen Y Gu J . fastp: An ultra-fast all-in-one FASTQ preprocessor. Bioinformatics. (2018) 34:i884–90. doi: 10.1093/bioinformatics/bty560 30423086 PMC6129281

[32] Patro R Duggal G Love MI Irizarry RA Kingsford C . Salmon provides fast and bias-aware quantification of transcript expression. Nat Methods. (2017) 14:417–9. doi: 10.1038/nmeth.4197 28263959 PMC5600148

[33] Soneson C Love MI Robinson MD . Differential analyses for RNA-seq: Transcript-level estimates improve gene-level inferences. F1000Res. (2015) 4:1521. doi: 10.12688/f1000research.7563.2 26925227 PMC4712774

[34] Love MI Huber W Anders S . Moderated estimation of fold change and dispersion for RNA-seq data with DESeq2. Genome Biol. (2014) 15:550. doi: 10.1186/s13059-014-0550-8 25516281 PMC4302049

[35] Braun A Dewert N Brunnert F Schnabel V Hardenberg J-H Richter B . Integrin αE(CD103) is involved in regulatory T-cell function in allergic contact hypersensitivity. J Invest Dermatol. (2015) 135:2982–91. doi: 10.1038/jid.2015.287 26203637

[36] Lucca LE Axisa P-P Singer ER Nolan NM Dominguez-Villar M Hafler DA . TIGIT signaling restores suppressor function of Th1 Tregs. JCI Insight. (2019) 4:e124427. doi: 10.1172/jci.insight.124427 30728325 PMC6413794

[37] Zhou L Lopes JE Chong MMW Ivanov II Min R Victora GD . TGF-beta-induced Foxp3 inhibits T(H)17 cell differentiation by antagonizing RORgammat function. Nature. (2008) 453:236–40. doi: 10.1038/nature06878 18368049 PMC2597437

[38] Deaglio S Dwyer KM Gao W Friedman D Usheva A Erat A . Adenosine generation catalyzed by CD39 and CD73 expressed on regulatory T cells mediates immune suppression. J Exp Med. (2007) 204:1257–65. doi: 10.1084/jem.20062512 17502665 PMC2118603

[39] Yue X Lio C-W Samaniego-Castruita D Li X Rao A . Loss of TET2 and TET3 in regulatory T cells unleashes effector function. Nat Commun. (2019) 10:2011. doi: 10.1038/s41467-019-09541-y 31043609 PMC6494907

[40] Chen S Zhang L Ying Y Wang Y Arnold PR Wang G . Epigenetically modifying the Foxp3 locus for generation of stable antigen-specific Tregs as cellular therapeutics. Am J Transplant. (2020) 20:2366–79. doi: 10.1111/ajt.15845 32167228 PMC7483360

[41] Kasahara H Okamoto S Sekiya T Yoshimura A . Vitamin C stabilizes Foxp3 expression in induced Treg cells by targeted DNA demethylation and prevents murine model of acute graft versus host disease. Blood. (2017) 130:70. doi: 10.1182/blood.V130.Suppl_1.70.70

[42] Lipsitz YY Timmins NE Zandstra PW . Quality cell therapy manufacturing by design. Nat Biotechnol. (2016) 34:393–400. doi: 10.1038/nbt.3525 27054995

[43] Lamarche C Ward-Hartstonge K Mi T Lin DTS Huang Q Brown A . Tonic-signaling chimeric antigen receptors drive human regulatory T cell exhaustion. Proc Natl Acad Sci USA. (2023) 120:e2219086120. doi: 10.1073/pnas.2219086120 36972454 PMC10083618

[44] Sudarsanam H Buhmann R Henschler R . Influence of culture conditions on ex vivo expansion of T lymphocytes and their function for therapy: Current insights and open questions. Front Bioeng Biotechnol. (2022) 10:886637. doi: 10.3389/fbioe.2022.886637 35845425 PMC9277485

[45] Tang Q Lee K . Regulatory T-cell therapy for transplantation: How many cells do we need? Curr Opin Organ Transplant. (2012) 17:349. doi: 10.1097/MOT.0b013e328355a992 22790069

[46] Polansky JK Kretschmer K Freyer J Floess S Garbe A Baron U . DNA methylation controls Foxp3 gene expression. Eur J Immunol. (2008) 38:1654–63. doi: 10.1002/eji.200838105 18493985

[47] Hippen KL Merkel SC Schirm DK Nelson C Tennis NC Riley JL . Generation and large-scale expansion of human inducible regulatory T cells that suppress graft-versus-host disease. Am J Transplant. (2011) 11:1148–57. doi: 10.1111/j.1600-6143.2011.03558.x 21564534 PMC3552455

[48] Zong Y Deng K Chong WP . Regulation of Treg cells by cytokine signaling and co-stimulatory molecules. Front Immunol. (2024) 15. doi: 10.3389/fimmu.2024.1387975 38807592 PMC11131382

[49] Ravindranath MH El Hilali F Filippone EJ . The impact of inflammation on the immune responses to transplantation: Tolerance or rejection? Front Immunol. (2021) 12:667834. doi: 10.3389/fimmu.2021.667834 34880853 PMC8647190

[50] Pirenne J Pirenne-Noizat F de Groote D Vrindts Y Lopez M Gathy R . Cytokines and organ transplantation. A review. Nucl Med Biol. (1994) 21:545–55. doi: 10.1016/0969-8051(94)90076-0 9234312

[51] Valencia X Stephens G Goldbach-Mansky R Wilson M Shevach EM Lipsky PE . TNF downmodulates the function of human CD4+CD25hi T-regulatory cells. Blood. (2006) 108:253–61. doi: 10.1182/blood-2005-11-4567 16537805 PMC1895836

[52] Gao Y Tang J Chen W Li Q Nie J Lin F . Inflammation negatively regulates FOXP3 and regulatory T-cell function via DBC1. Proc Natl Acad Sci. (2015) 112:E3246–54. doi: 10.1073/pnas.1421463112 26060310 PMC4485127

[53] Feldhoff LM Rueda CM Moreno-Fernandez ME Sauer J Jackson CM Chougnet CA . IL-1β induced HIF-1α inhibits the differentiation of human FOXP3+ T cells. Sci Rep. (2017) 7:465. doi: 10.1038/s41598-017-00508-x 28352109 PMC5428734

[54] Yang XO Nurieva R Martinez GJ Kang HS Chung Y Pappu BP . Molecular antagonism and plasticity of regulatory and inflammatory T cell programs. Immunity. (2008) 29:44–56. doi: 10.1016/j.immuni.2008.05.007 18585065 PMC2630532

[55] Wing K Onishi Y Prieto-Martin P Yamaguchi T Miyara M Fehervari Z . CTLA-4 control over Foxp3+ regulatory T cell function. Science. (2008) 322:271–5. doi: 10.1126/science.1160062 18845758

[56] Roberts V Stagg J Dwyer KM . The role of ectonucleotidases CD39 and CD73 and adenosine signaling in solid organ transplantation. Front Immunol. (2014) 5. doi: 10.3389/fimmu.2014.00064 24600452 PMC3927137

[57] Joller N Lozano E Burkett PR Patel B Xiao S Zhu C . Treg cells expressing the coinhibitory molecule TIGIT selectively inhibit proinflammatory Th1 and Th17 cell responses. Immunity. (2014) 40:569–81. doi: 10.1016/j.immuni.2014.02.012 24745333 PMC4070748

[58] Hartigan CR Tong KP Liu D Laurie SJ Ford ML . TIGIT agonism alleviates costimulation blockade resistant rejection in a Treg-dependent manner. Am J Transplant. (2023) 23:180–9. doi: 10.1016/j.ajt.2022.12.011 36695691 PMC10062175

[59] Yan J Zhang Y Zhang J-P Liang J Li L Zheng L . Tim-3 expression defines regulatory T cells in human tumors. PloS One. (2013) 8:e58006. doi: 10.1371/journal.pone.0058006 23526963 PMC3589491

[60] Nieves-Rosado HM Banerjee H Gocher-Demske A Manandhar P Mehta I Ezenwa O . Tim-3 is required for regulatory T cell-mediated promotion of T cell exhaustion and viral persistence during chronic lymphocytic choriomeningitis virus infection. J Immunol. (2024) 213:1488–98. doi: 10.4049/jimmunol.2400119 39345172 PMC11671103

[61] Banerjee H Nieves-Rosado H Kulkarni A Murter B McGrath KV Chandran UR . Expression of Tim-3 drives phenotypic and functional changes in Treg cells in secondary lymphoid organs and the tumor microenvironment. Cell Rep. (2021) 36:109699. doi: 10.1016/j.celrep.2021.109699 34525351 PMC8482289

[62] Banerjee H Kane LP . Immune regulation by tim-3. F1000Res. (2018) 7:316. doi: 10.12688/f1000research.13446.1 29560265 PMC5854983

[63] Ferris RL Lu B Kane LP . Too much of a good thing? Tim-3 and TCR signaling in T cell exhaustion. J Immunol. (2014) 193:1525–30. doi: 10.4049/jimmunol.1400557 25086175 PMC4120324

[64] Lamarche C Levings MK . Guiding regulatory T cells to the allograft. Curr Opin Organ Transplant. (2018) 23:106. doi: 10.1097/MOT.0000000000000483 29140829

[65] Zhang N Schröppel B Lal G Jakubzick C Mao X Chen D . Regulatory T cells sequentially migrate from inflamed tissues to draining lymph nodes to suppress the alloimmune response. Immunity. (2009) 30:458–69. doi: 10.1016/j.immuni.2008.12.022 19303390 PMC2737741

[66] Lee I Wang L Wells AD Dorf ME Ozkaynak E Hancock WW . Recruitment of Foxp3+ T regulatory cells mediating allograft tolerance depends on the CCR4 chemokine receptor. J Exp Med. (2005) 201:1037–44. doi: 10.1084/jem.20041709 15809349 PMC2213137

[67] Hoerning A Köhler S Jun C Tebbe B Fu J Menke J . Peripherally circulating CD4^+^ FOXP3^+^ CXCR3^+^ T regulatory cells correlate with renal allograft function. Scand J Immunol. (2012) 76:320–8. doi: 10.1111/j.1365-3083.2012.02732.x 22670785 PMC3425730

[68] Contreras-Castillo E García-Rasilla VY García-Patiño MG Licona-Limón P . Stability and plasticity of regulatory T cells in health and disease. J Leukoc Biol. (2024) 116:33–53. doi: 10.1093/jleuko/qiae049 38428948

[69] Diller ML KudChadkar RR Delman KA Lawson DH Ford ML . Balancing inflammation: The link between Th17 and regulatory T cells. Mediators Inflammation. (2016) 2016:6309219. doi: 10.1155/2016/6309219 27413254 PMC4930807

[70] Koenecke C Lee C-W Thamm K Föhse L Schafferus M Mittrücker H-W . IFN-γ production by allogeneic Foxp3+ regulatory T cells is essential for preventing experimental graft-versus-host disease. J Immunol. (2012) 189:2890–6. doi: 10.4049/jimmunol.1200413 22869903

[71] Okamoto M Kuratani A Okuzaki D Kamiyama N Kobayashi T Sasai M . IFN-γ-induced Th1-Treg polarization in inflamed brains limits exacerbation of experimental autoimmune encephalomyelitis. Proc Natl Acad Sci USA. (2024) 121:e2401692121. doi: 10.1073/pnas.2401692121 39560646 PMC11621829

[72] Song MH Nair VS Oh KI . Vitamin C enhances the expression of IL17 in a Jmjd2–dependent manner. BMB Rep. (2017) 50:49–54. doi: 10.5483/BMBRep.2017.50.1.193 27931518 PMC5319665

[73] Kopf H de la Rosa GM Howard OMZ Chen X . Rapamycin inhibits differentiation of Th17 cells and promotes generation of FoxP3+ T regulatory cells. Int Immunopharmacol. (2007) 7:1819–24. doi: 10.1016/j.intimp.2007.08.027 17996694 PMC2223142

[74] Ferreira LMR Muller YD Bluestone JA Tang Q . Next-generation regulatory T cell therapy. Nat Rev Drug Discov. (2019) 18:749–69. doi: 10.1038/s41573-019-0041-4 31541224 PMC7773144

[75] Kouakanou L Peters C Sun Q Floess S Bhat J Huehn J . Vitamin C supports conversion of human γδ T cells into FOXP3-expressing regulatory cells by epigenetic regulation. Sci Rep. (2020) 10:6550. doi: 10.1038/s41598-020-63572-w 32300237 PMC7162875

[76] Yue Y Ren Y Lu C Li P Zhang G . Epigenetic regulation of human FOXP3+ Tregs: From homeostasis maintenance to pathogen defense. Front Immunol. (2024) 15:1444533. doi: 10.3389/fimmu.2024.1444533 39144146 PMC11323565

[77] Chen J Guo L Zhang L Wu H Yang J Liu H . Vitamin C modulates TET1 function during somatic cell reprogramming. Nat Genet. (2013) 45:1504–9. doi: 10.1038/ng.2807 24162740

[78] Issuree PD Teghanemt A Heath J Thurman A Pezzulo A . DNA demethylation fine-tunes IL-2 production during thymic development and promotes regulatory T cell differentiation. J Immunol. (2023) 210:76.24. doi: 10.4049/jimmunol.210.Supp.76.24 PMC10157375 36880575

[79] Gawronski M Starczak M Wasilow A Dziaman T Olinski R Gackowski D . Loss of TET2 activity limits the ability of vitamin C to activate DNA demethylation in human HAP1 cells. Epigenet Chromatin. (2025) 18:76. doi: 10.1186/s13072-025-00634-1 41287077 PMC12642257

[80] Kasahara H Kondo T Nakatsukasa H Chikuma S Ito M Ando M . Generation of allo-antigen-specific induced Treg stabilized by vitamin C treatment and its application for prevention of acute graft versus host disease model. Int Immunol. (2017) 29:457–69. doi: 10.1093/intimm/dxx060 29126272

