# Supplementary figures and images for: Increased phenotypic and functional stability of human allospecific induced Tregs is associated with Vitamin C-mediated FOXP3 TSDR demethylation
Source: Front Immunol. 2026 Jul 8;17:1827886. doi: 10.3389/fimmu.2026.1827886 (PMC13388274; doi:10.3389/fimmu.2026.1827886)

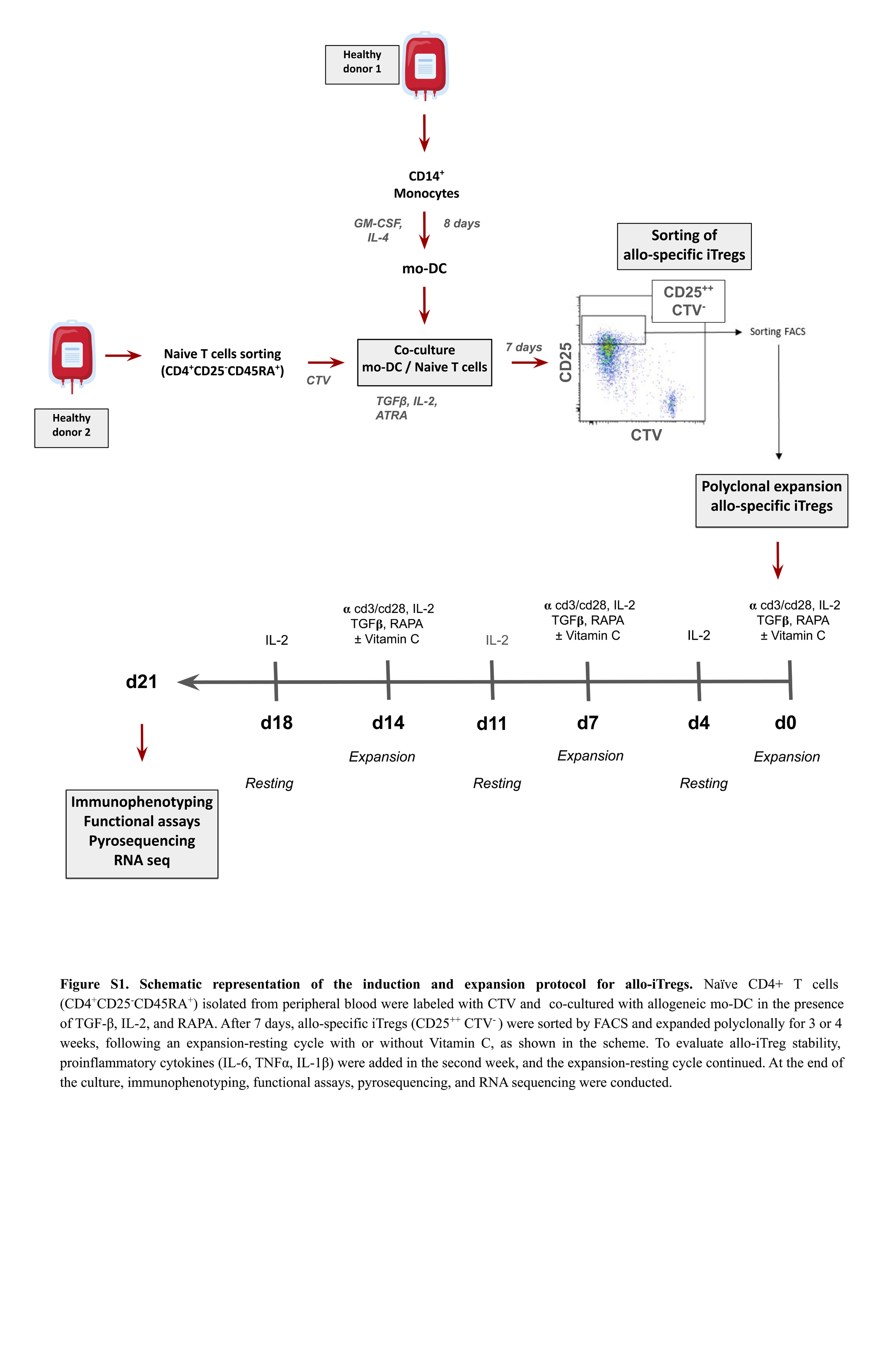

Supplement: Supplementary file 1 [file Image1.tif]

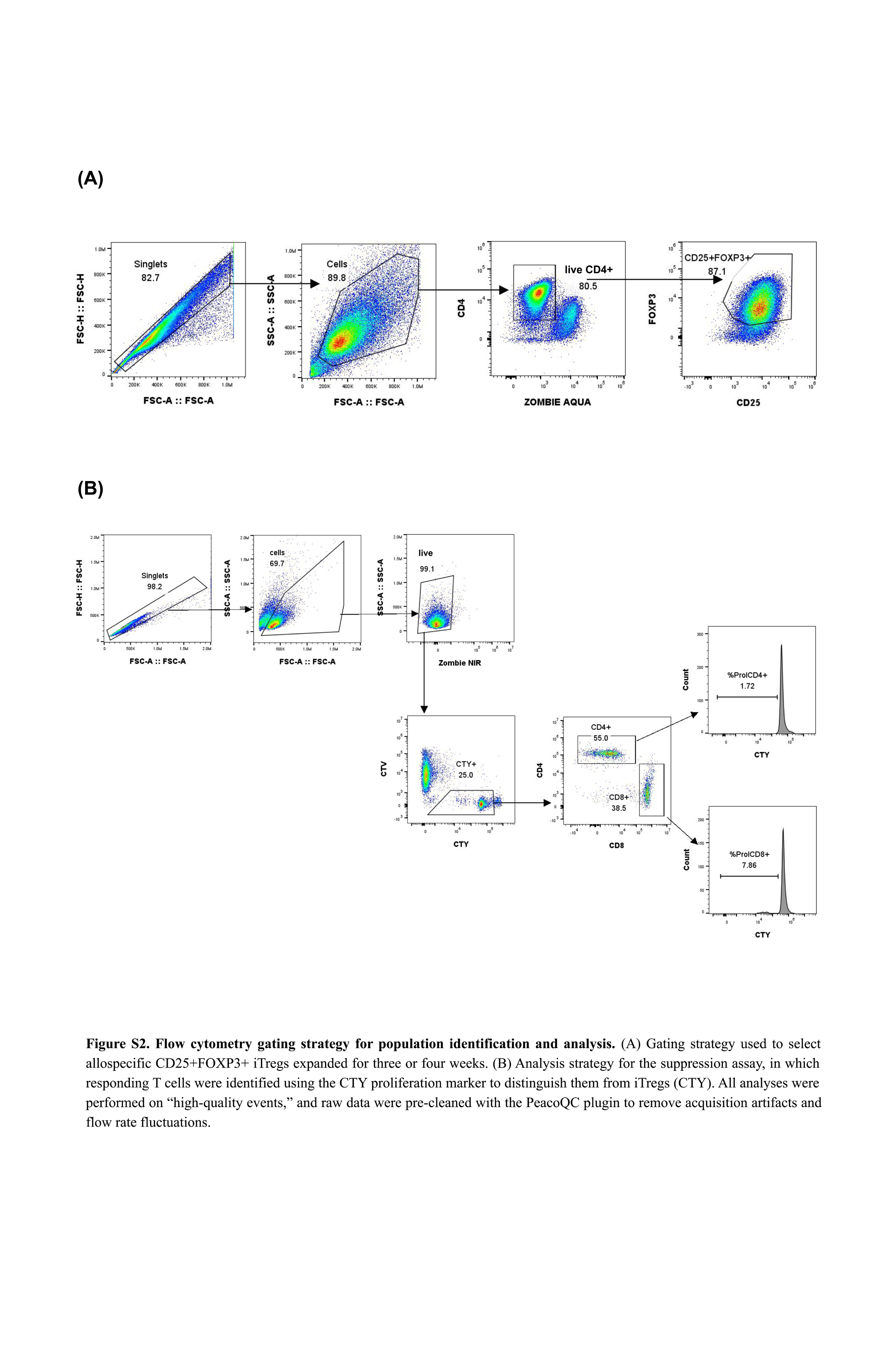

Supplement: Supplementary file 2 [file Image2.tif]

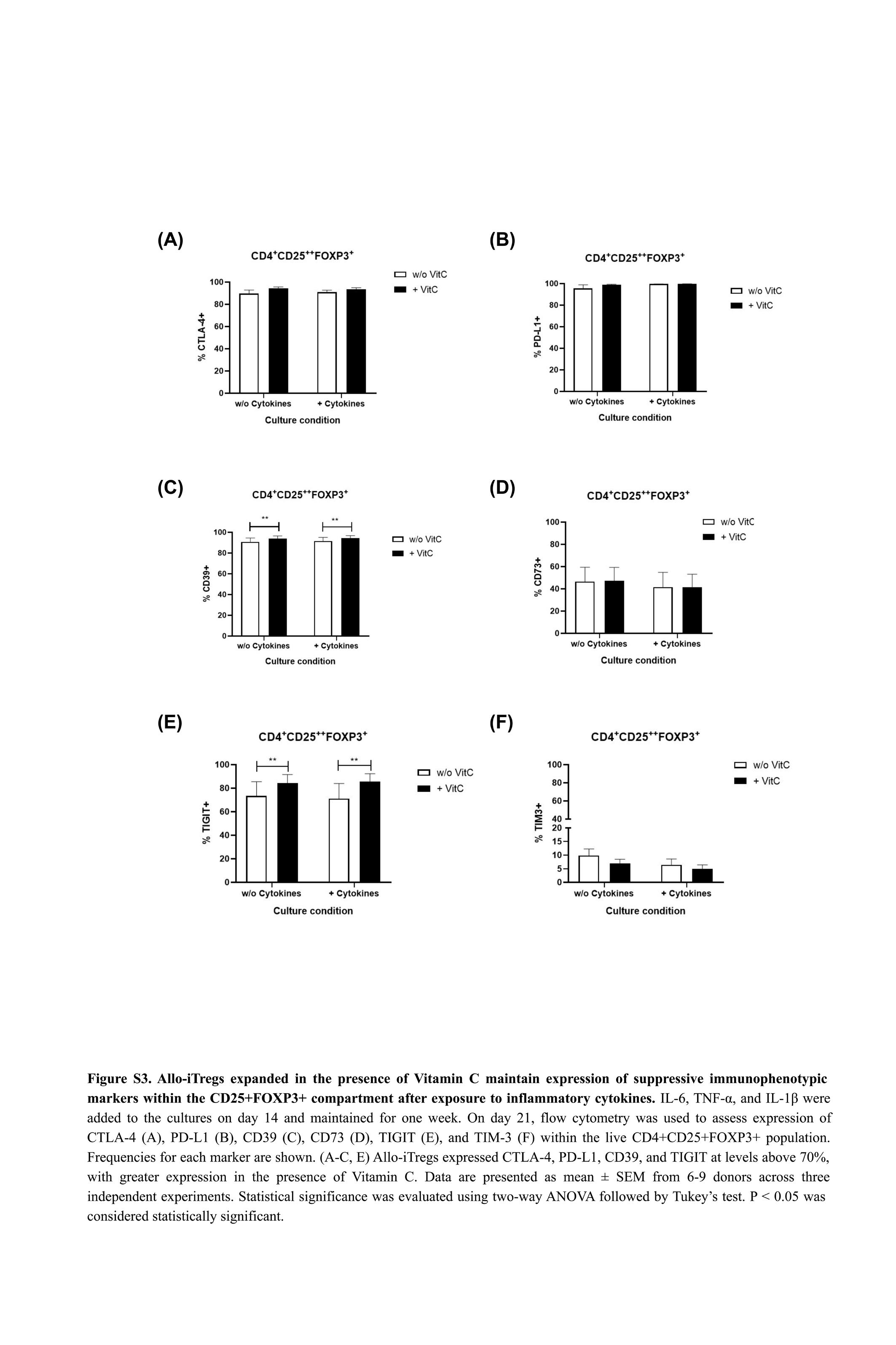

Supplement: Supplementary file 3 [file Image3.tif]

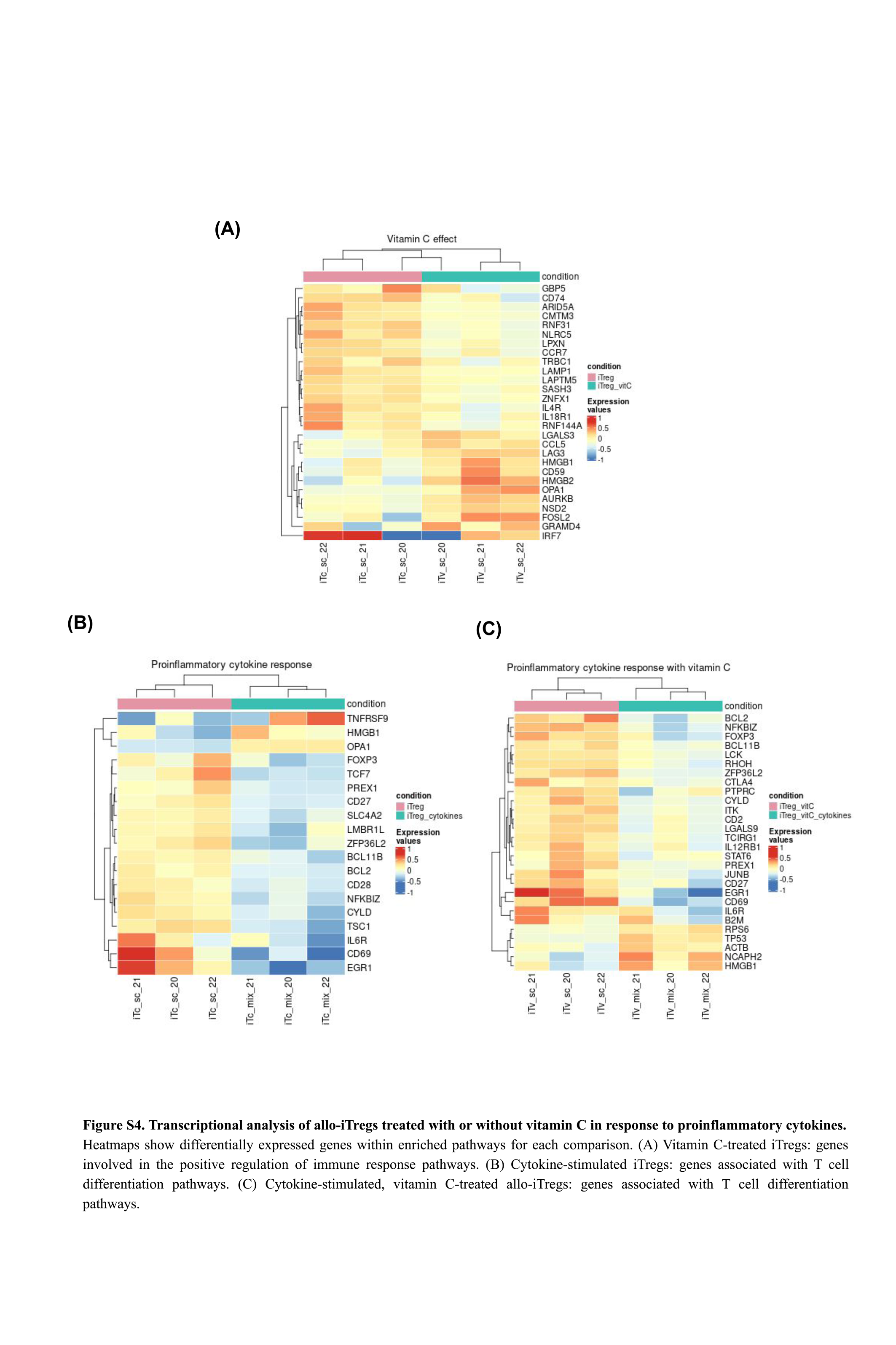

Supplement: Supplementary file 4 [file Image4.tif]
