# Supplementary material for: Increased phenotypic and functional stability of human allospecific induced Tregs is associated with Vitamin C-mediated FOXP3 TSDR demethylation
Source: Front Immunol. 2026 Jul 8;17:1827886. doi: 10.3389/fimmu.2026.1827886 (PMC13388274; doi:10.3389/fimmu.2026.1827886)
Supplement: Supplementary file 5 [file Table1.docx]

TABLE 1

| **CpG Sites** | **Primer sequence** | **PCR conditions** | **Size bp** |
| --- | --- | --- | --- |
|  | **Set I** |  |  |
| 1-3 | Forward: 5´-ATTTGTTTGGGGGTAGAG-3´ | Step 1:  95 °C for 15 min  Step 2: 30 cycles  95 °C for 30 s  51.1 °C for 30 s  72 °C for 30 s  Step 3:  72 °C for 5 min | 170 |
|  | Reverse (Biotin): 5´-TATAACAACAAAACCCAAATAC-3´ |  |  |
|  | Sequence: 5´-GTAGAGGATTTAGAGGG-3´ |  |  |
|  | Analysis Sequence: 5´T**Y**GGGTTGGGTAGT**Y**GGTTTTTTGTATTGTTTGTTGGGA**Y**GTTTTTTTTTGATTGGGTTTTTTAGAAG-3´ |  |  |
|  |  |  |  |
|  | **Set II A and B** |  |  |
| 4-5 | Forward: 5´-GGATGTTTTTGGGATATAG-3´ | Step 1:  95 °C for 15 min  Step 2: 15 cycles  95 °C for 30 s  56 °C decrease 0.5 °C per cycle for 30 s  72 °C for 20 s  Step 3: 20 cycles  95 °C for 30 s  49 °C for 30 s  72 °C for 20 s  Step 4:  72 °C for 5 min | 167 |
|  | Reverse (Biotin): 5´AATAAAATATCTACCCTCTTCTCT-3´ |  |  |
|  | Sequence 1: 5´-GATGTTTTTGGGATATAGATTA |  |  |
|  | Analysis Sequence A: 5´TGTTTTTATAT**Y**GGGGTTTGTATTTGGGTTTTGTTGTTATAGTTTT**Y**GATTTGTTTAGATTTT3´ |  |  |
| 6-12 | Sequence 2: 5´-GATTTGTTAGATTTT-3´ |  |  |
|  | Analysis Sequence B:  5´TT**Y**GTTATTGA**Y**GTTATGG**Y**GGT**Y**GGATG**Y**GT**Y**GGGTTTTATCGATATTA**Y**GGAGGAAGAGAAGAGGGTAGATATTTTA-3´ |  |  |
|  |  |  |  |
|  | **Set III** |  |  |
|  | Forward: 5´-GAGGAAGAGAAGAGGGTAGATA-3´ | Step 1:  95 °C for 15 min  Step 2: 15 cycles  95 °C for 30 s  60 °C decrease 0.5 °C per cycle for 30 s  72 °C for 20 s  Step 3: 20 cycles  95 °C for 30 s  53 °C for 30 s  72 °C for 20 s  Step 4:  72 °C for 5 min | 107 |
|  | Reverse (Biotin): 5´-CACCAACACCCATATCAC-3´ |  |  |
| 13-14 | Sequence: 5´AGGGTAGATATTTTATTTTATAGG-3´ |  |  |
|  | Analysis Sequence:  5´TTT**Y**GTTT**Y**GAGAATTGGTTGTTTTGTTTTGTAGTA-3´ |  |  |

Supplementary Table: Primers and PCR conditions for pyrosequencing analysis.
